# A microscale thermophoresis-based enzymatic RNA methyltransferase assay enables the discovery of DNMT2 inhibitors

**DOI:** 10.1038/s42004-025-01439-9

**Published:** 2025-02-03

**Authors:** Zarina Nidoieva, Mark O. Sabin, Tristan Dewald, Annabelle C. Weldert, Sabrina N. Hoba, Mark Helm, Fabian Barthels

**Affiliations:** 1 Institute of Pharmaceutical and Biomedical Sciences, Johannes Gutenberg-University, Mainz, Germany; 2 Department of Molecular, Cellular, and Developmental Biology, Yale University, New Haven, CT USA

**Keywords:** Nucleic acids, Chemical tools, Target identification

## Abstract

RNA methyltransferases (MTases) have recently become increasingly important in drug discovery. Yet, most frequently utilized RNA MTase assays are limited in their throughput and hamper this rapidly evolving field of medicinal chemistry. This study developed a microscale thermophoresis (MST)-based split aptamer assay for enzymatic MTase investigations, improving current methodologies by offering a non-proprietary, cost-effective, and highly sensitive approach. Our findings demonstrate the assay’s effectiveness across different RNA MTases, including inhibitor characterization of METTL3/14, DNMT2, NSUN2, and *S. aureus* TrmD, enabling future drug discovery efforts. Using this concept, a pilot screening on the cancer drug target DNMT2 discovered several hit compounds with micromolar potency.

## Abstract (web summary)

RNA methyltransferases (MTases) are drug targets that catalyze RNA methylation, a reaction associated to disease, but medium and high-throughput screening assays are lacking. Here, the authors develop a microscale thermophoresis-based split aptamer assay and demonstrate its sensitivity and robustness in the characterization of inhibitors against RNA MTases.

## Introduction

RNA has recently emerged in modern drug discovery, serving not only as a drug itself but also as a target for drugs, and notably, as a central component in cellular pathways linked to malignancies and infections^1,2^. Methylation of RNA, a metabolic process observed across all organisms, has been validated for its association with numerous diseases^3^. In this regard, the introduction of STC-15, the first-in-class RNA methyltransferase (MTase) inhibitor targeting METTL3/14, into clinical trials, marks a significant paradigm shift^4^. Thus, detecting and quantifying RNA modifications, including nucleobase methylation, has become a focal point of research due to their physiological importance and implication in diseases. The current gold-standard bioanalytical techniques encompass mass spectrometry, radiometry, and sequencing methods^5^. Yet, the detection of *S*-adenosylhomocysteine (SAH), a co-product of the MTase reactions, offers a simpler and universal assay methodology^6^.

Enzymatic scintillation assays using ^3^H-SAM and the LC/MS-based detection of the MTase reaction co-product SAH are state-of-the-art for screening drug candidates on RNA MTases such as METTL3/14^7^, DNMT2^8^, NSUN2^9^, and anti-infective targets like *H. influenzae* TrmD or SARS-CoV2 nsp10/16^10,11^. Despite these advancements, existing RNA MTase assays face limitations in throughput, impeding the rapid progress of medicinal chemistry in this domain. Efforts to overcome these limitations include the development of antibodies capable of discriminating between SAH and *S*-adenosylmethionine (SAM)^12^. However, these antibodies lack the necessary affinity and specificity for robust SAH detection at low concentrations. Indirect SAH detection via enzyme-coupled assays is another approach, yet none possess the requisite sensitivity for low nanomolar range detection^13–16^. Methyltransferases present numerous challenges in enzymatic assay development due to their slow kinetic nature and substrate affinity in the sub-micromolar range^17^. This necessitates highly sensitive enzymatic assay methods, requiring the detection of SAH at very low concentrations (<50 nM). Very recently, Pham et al. used an SAH-binding RNA aptamer to develop a time-resolved fluorescence energy transfer (TR-FRET)-based SAH sensor which is now sold commercially as “AptaFluor™ SAH Methyltransferase Assay Kit”^18^. While this approach is superior to previous assay concepts in terms of sensitivity and robustness, it is marketed proprietary (i.e., does not reveal the aptamer RNA sequences) and relatively high cost per treatment condition ($5/single assay reaction) still hampering high- or even medium-throughput assay campaigns in academic settings.

In this study, we followed a similar approach to develop an aptamer-based RNA MTase assay, aiming to address the major drawbacks of previous methods. We extend the utility of the assay beyond TR-FRET readers to conventional FRET-compatible plate readers or microscale thermophoresis (MST) instruments. Hence, the development of an MST protocol for RNA methylation quantification offers practical advantages over earlier technologies, potentially enabling RNA MTase drug discovery campaigns previously deemed unfeasible. Our protocol is non-proprietary, revealing all necessary RNA aptamer sequences for independent assay setup and protocol modification at a moderate expense (<$0.05/single assay reaction).

## Results and discussion

### Design and calibration of a FRET- and MST-capable SAH-split aptamer

To engineer a SAH-binding aptamer for RNA MTase enzyme assay development, we chose a naturally occurring 68-nucleotide RNA aptamer identified previously upstream of the metH gene in *D. aromatica* that is part of a riboswitch, controlling metH expression depending on the SAH abundance in bacteria^19^. RNA aptamers are highly structured polynucleotides that form selective binding pockets for specific ligands^20^. While the first aptamers were developed by in vitro selection approaches, structures with similar properties were later discovered in nature, most often in bacteria, engineered aptamers can be developed to exhibit high affinity and selectivity for their targets, and can have considerable potential for applications in biotechnology^21^. This metH aptamer represents the shortest consensus sequence within a group of sequences from related organisms, as it lacks an optional structural element termed P3 stem. The apparent dissociation constant *K*_D_ of this metH aptamer for SAH was determined by isothermal titration calorimetry to be around 20 nM with at least 100-fold selectivity over other nucleosides (including SAM) highlighting the suitability for our assay development campaign^22^.

**Fig. 1:**
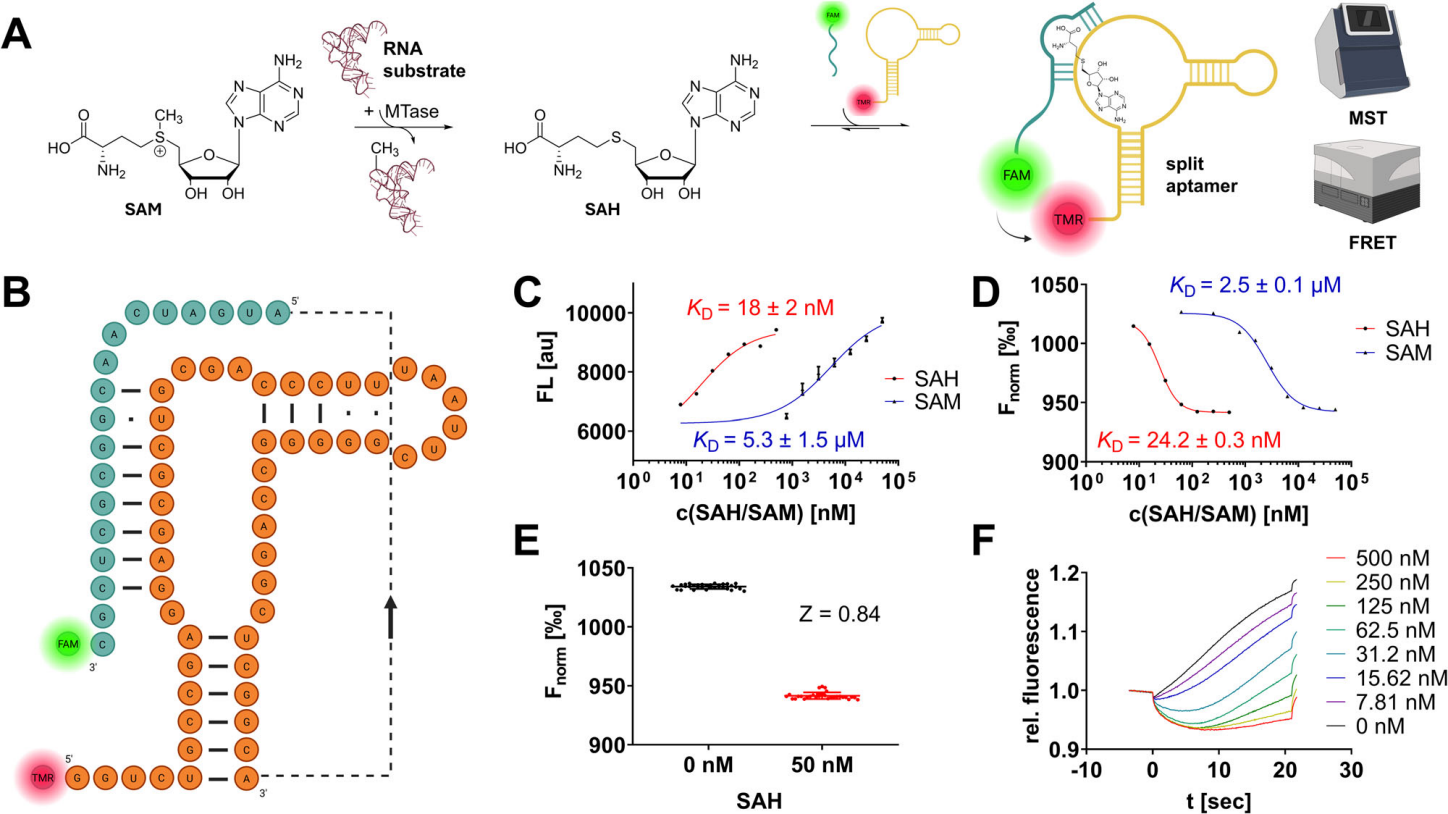
Development and calibration of an SAH-sensing split aptamer. **A** Schematic presentation of the assay concept: The universal RNA MTase co-product SAH binds to a FAM/TAMRA labeled split aptamer which can be investigated by microscale thermophoresis (MST) and FRET experimental setups. **B** Sequence and secondary structure model of the split aptamer consisting of 5′-TAMRA labeled aptaSAH1 (orange) and 3′-FAM labeled aptaSAH2 (cyan). **C** Dose-response curves of aptaSAH1&2 (each 50 nM) in the presence of the SAH or SAM ligand by plate reader-based FRET assays (λ_ex_ = 485 nm; λ_em_ = 600 nm). **D** Dose-response curves analogously to subfigure (**C**) but determined from MST experiments with variable concentrations of SAH and SAM titrated to the aptamer highlight the dynamic range of the assay scope (mean ± SD, *n* = 3). **E** Z-factor determination at 50 nM SAH (*n* = 32) to evaluate the robustness of the assay setup. **F** MST traces of aptaSAH1&2 (each 50 nM) in the presence of the SAH ligand yielded significant thermophoresis shifts.

In our study, we followed the aim of developing a fluorescent split aptamer SAH biosensor (Fig. 1A) from the natural non-split metH RNA aptamer. The concept of fluorescent split aptamers relies on a series of two or more independent fluorescently labeled aptamer oligonucleotides, able to assemble only in the presence of a specific target ligand. In a FRET-based approach, two fragments carry a FRET-pair of communicating fluorescent dyes, such that, upon excitation of the donor dye, the acceptor dye will only emit fluorescence if the dyes are in spatial proximity in the ligand-bound complex, thus indicating the presence of the analyte, i.e., SAH^23^. We hypothesized a suitable site for splitting the metH aptamer at adenine-50 from previously reported in-line probing and crystallography experiments on SAH riboswitches^19,22^, hence, we will describe this specific design and sequences. Accordingly, we evaluated two split aptamer fragments labeled with fluorescein (FAM) and tetramethylrhodamin (TAMRA) as a FRET-capable dye pair: aptaSAH1 and aptaSAH2 (Fig. 1B).

To establish our experimental setup, we mixed both aptamer fragment oligonucleotides at equimolar concentrations (final: 50 nM) and titrated series of SAH resp. SAM dilutions to this prototypical SAH biosensor. FRET experiments (λ_ex_ = 485 nm; λ_em_ = 600 nm) in 96-well plates yielded concentration-dependent fluorescence read-outs with apparent *K*_D_ values for SAH and SAM of 18 nM and 5.3 µM in agreement with the native full-length metH aptamer affinities (Fig. 1C)^19^. Hence, this 300-fold SAH over SAM selectivity and nanomolar sensitivity provides the necessary framework for the development of an enzymatic methyltransferase assay. Yet, we tested the same experimental setup with an MST-based acquisition (Fig. 1D, F) which revealed comparable *K*_D_ values (24 nM & 2.5 µM) but significantly improved reproducibility and signal-to-noise properties (S/N_MST_ = 41 dB vs. S/N_FRET_ = 33 dB). Furthermore, MST assays require only 10 µL of sample volume per capillary compared to 30 µL per well in a 96-well plate supporting high-throughput applications in material consumption.

To assess the suitability for sensing SAH in a steady-state enzymatic assay, we examined statistical Z-values at 50 nM SAH (which correspond to 5% SAM-to-SAH substrate conversion from 1 µM SAM substrate in a common RNA MTase reaction). By this, we determined an MST-based Z-value of 0.84 (FRET assay: *Z* = 0.57) highlighting the suitability of this framework for high-throughput screening (HTS) assay setups (Fig. 1E). For these reasons, we prefer the usage of the MST-based assay which the following chapter will focus on, but it is noteworthy that plate reader-based FRET assays were also found equally functional. Furthermore, we performed comparative assays with the commercial AptaFluor assay kit, which gave similar results to our assay in all respects (SI Fig. 2): Z-factor at 50 nM SAH = 0.84; SAH resp. SAM affinity *(K*_D_ = 26 nM & 5.7 µM).

### MST-based assays for the characterization of RNA MTase activity and functional inhibitory and enzymatic parameters

**Fig. 2:**
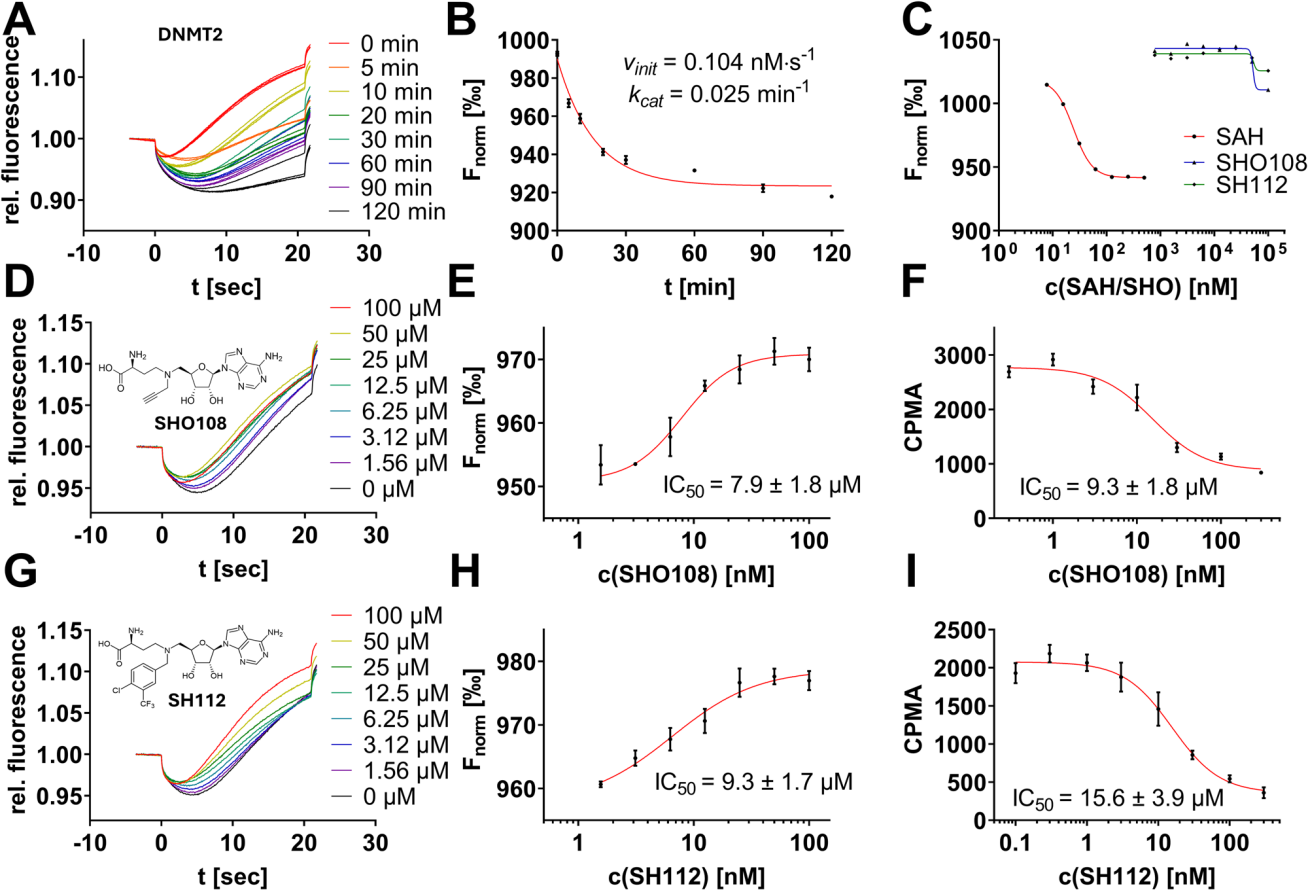
DNMT2 enzyme assays to characterize the suitability of the split aptamer to investigate RNA MTase kinetics. **A** MST traces of the DNMT2 enzyme reaction (250 nM DNMT2, 5 µM tRNA, 0.9 µM SAM) incubated for variable durations (0–120 min) and yielded significant thermophoresis shifts. **B** Substrate conversion plots (F_norm_ vs incubation time) reveal steady-state DNMT2 kinetics. **C** Dose-response curves of aptaSAH1&2 (both 50 nM) in the presence of SHO108/SH112 inhibitors (0–100 µM) indicated these SAH-analogs are not binding to the aptamer at relevant concentrations. **D**–**F** Inhibitor characterization of SHO108 from left to right: MST traces at variable inhibitor concentrations; MST-derived dose-response curves; orthogonal ^3^H-incorporation assay-derived dose-response curves from detected counts per minute (CPMA). **G**–**I** Inhibitor characterization of SH112 analogous to subplot (**D**–**F**). All inhibitor characterizations were performed in triplicates (mean ± SD, *n* = 3).

In this manuscript, we aimed to validate a split aptamer assay setup by reproducing functional enzyme assay results using enzyme kinetics of RNA MTases and their known inhibitors. The enzyme kinetics were characterized by calculating the Michaelis-Menten constant (*K*_M_ or *k*_*cat*_) and inhibitory constants (IC_50_ resp. *K*_I_). First, we focused on the characterization of human DNMT2, a well-characterized model of m^5^C methyltransferase, using its known enzyme kinetics and inhibitors^24^. To assess DNMT2 activity, we incubated 250 nM DNMT2 with 5 µM tRNA, and 0.9 µM SAM for variable durations (0–120 min) and probed with the split aptamer and MST-based evaluation for the SAH content formed during the reaction (Fig. 2A). The production of total SAH (in nM) could be calculated from the respective F_norm_ values (Fig. 2B) and regression to the calibration curve in Fig. 1D, indicating the enzymatic reaction reaches a plateau after 30 min when approximately 10% SAM consumption was reached (112 nM SAH from 900 nM SAM), consistent with its intrinsic product inhibition reported in the literature^25^. The initial reaction velocity (*v*_*init*_) was determined to be 0.104 nM s^−1^, and the turnover number (*k*_*cat*_) was calculated as 0.025 min^−1^.

To evaluate the effect of inhibitors on DNMT2 activity, we used two known inhibitors, SHO108 and SH112. These SAH derivatives were selected based on their documented efficacy for DNMT2^8,26^. Before setting up an enzyme assay experiment, both inhibitors (without enzyme or RNA substrate) were titrated up to 100 µM to the split aptamer revealing only minimal MST shifts compared to the SAH-positive control. This indicated that, although these inhibitors share structural features with the aptamer ligand SAH, the split aptamer provides sufficient selectivity for SAH over such derivates to allow inhibition experiments with the present assay setup (Fig. 2C). Additionally, the natural *D. aromatica* (non-split) SAH aptamer has been tested for binding of 16 SAH-like derivatives in previous studies, showing excellent selectivity for SAH, as all derivatives exhibited binding affinities at least 3 orders of magnitude weaker^19^. We followed this strategy, using our split aptamer construct, we tested 10 additional SAH derivatives at 10 µM among which only the SAH-mimetic and pan-MTase inhibitor sinefungin showed a slight MST shift in the presence of the split aptamer, highlighting the excellent selectivity for SAH (SI Fig. 3).

To further validate our findings on DNMT2, we compared the split aptamer inhibition assay results of both inhibitors with those obtained from a previously published orthogonal assay method, the radiometric ^3^H incorporation assay^8,26^. Dose-response experiments were conducted to measure inhibitory constants, demonstrating high reproducibility between the two methods (Fig. 2D–I). In summary, both SHO108 and SH112 inhibitors showed dose-dependent inhibition of DNMT2 activity with comparable inhibitory constants between orthogonal methods, demonstrating that the split aptamer assay could reproduce previously published results while circumventing the use of radioactivity. Using an additional non-enzymatic MTase assay technique, we also investigated the two DNMT2 inhibitors utilizing an orthogonal MST method based on a fluorescent tracer displacement assay (SI Fig. 1)^27,28^. SHO108 and SH112 showed *K*_D_ values of 4.7 and 7.8 µM, respectively, in good agreement with the inhibition data presented in Fig. 2.

**Fig. 3:**
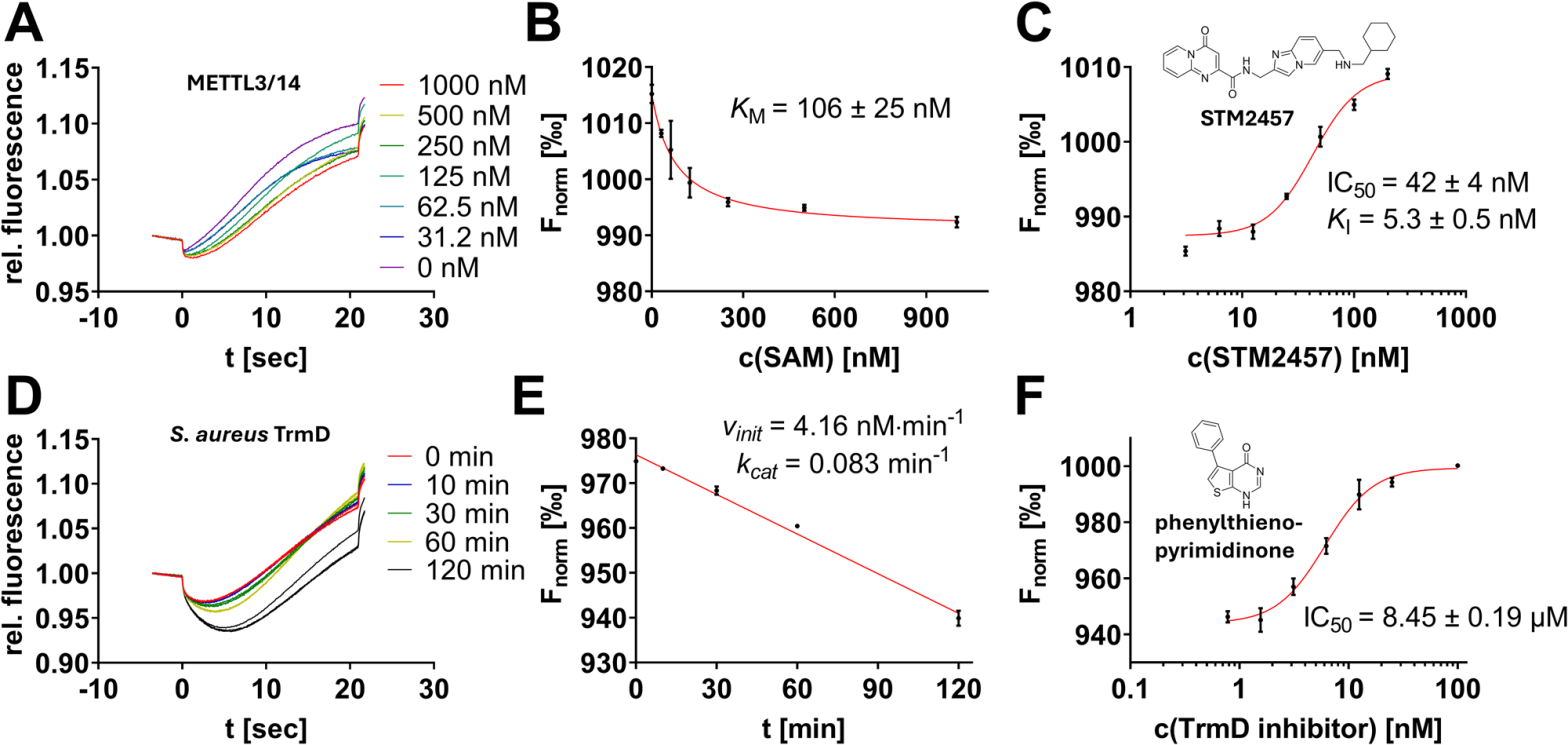
MTase enzyme assays to characterize METTL3/14 and *S. aureus* TrmD. **A**, **B** MST traces of the METTL3/14 enzyme reaction incubated with variable concentrations of SAM yielded significant thermophoresis shifts and enabled the determination of the Michaelis-Menten constant *K*_*M*_. **C** Inhibitor characterization of STM2457: MST-derived dose-response curves. **D**, **E** MST traces and substrate conversion plots (F_norm_ vs time) revealing initial *S. aureus* TrmD kinetics. **F** Inhibitor characterization of 5-phenylthieno[2,3-*d*]pyrimidin-4(1*H*)-one: MST-derived dose-response curves. All inhibitor characterizations were performed in triplicates (mean ± SD, *n* = 3).

These results validate the split aptamer assay setup for studying DNMT2 activity and inhibitor efficacy. Subsequently, we extended our investigation to include other RNA MTases of clinical relevance, employing diverse inhibitor scaffolds as reported in the literature. Specifically, we studied the m^6^A MTase METTL3/14 and the m^7^G MTase *S. aureus* TrmD. For METTL3/14 (10 nM MTase, 500 nM SAM, 120 nM RNA substrate), we could successfully reproduce a reported *K*_*M*_ value of 106 nM (Fig. 3A, B), demonstrating the assay’s capability in this context^29^. To further illustrate the assay’s utility for drug discovery, we also performed inhibition experiments with the known inhibitor STM2457, yielding an apparent inhibition constant *K*_I_ of 5.1 nM (Fig. 3C). This result is consistent with both an orthogonal METTL3/14 assay utilizing an anti-m^6^A-antibody and the mass-spectrometric characterization reported in the literature^7,29^, which confirms that our assay is also suitable for characterizing high-affinity inhibitors with low nanomolar potency.

Additionally, we investigated the bacterial m^7^G MTase *S. aureus* TrmD, for which anti-infective inhibitors have been developed^10^. Time-course experiments with 50 nM TrmD, 2 µM SAM, and 1 µM tRNA^Leu^ indicated that our assay is also appropriate for studying MTases with slow kinetics. TrmD exhibited a catalytic efficiency approximately one order of magnitude lower than DNMT2 and METTL3/14, with *v*_*init*_ and *k*_*cat*_ values of 4.16 nM min^–1^ and 0.083 min^–1^, respectively (Fig. 3D, E). Notably, the SAH production of TrmD does not reach saturation after 120 min. Furthermore, we could validate the assay’s applicability for drug discovery by confirming a known TrmD inhibitor with an IC_50_ of 8.45 µM, closely matching the reported value of 11 µM (Fig. 3F)^10^.

**Fig. 4:**
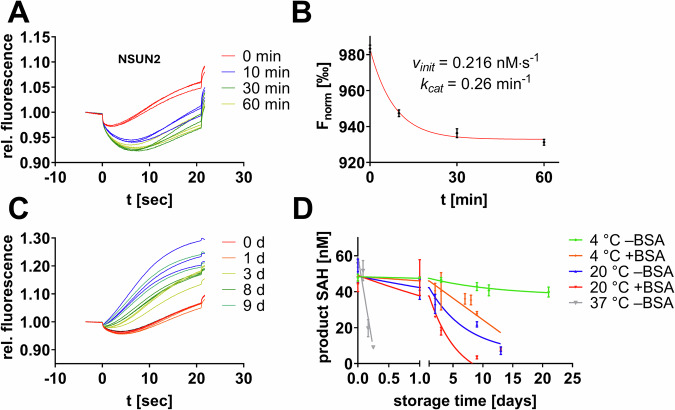
Enzyme stability assessment of the m^5^C MTase NSUN2 by aptamer-based MST experiments. **A** MST traces of the NSUN2 enzyme incubated for variable durations (0–60 min) yielded significant thermophoresis shifts. **B** Substrate conversion plots (F_norm_ vs time) reveal steady-state NSUN2 kinetics (mean ± SD, *n* = 3). **C** Exemplary set of MST traces of the NSUN2 enzyme incubated for variable durations in storage buffer containing 0.5% BSA (w/w) at 20 °C. **D** Assessment of NSUN2 stability by catalytic competence after a defined duration in storage buffer (with or without the addition of BSA) and storage at variable temperatures (mean ± SD, *n* = 3).

Moreover, we intended to use the split aptamer MST assay for a stability assessment study of the m^5^C MTase NSUN2 in order to develop conditions suitable for stabilizing NSUN2 for several days to perform drug discovery screening applications in the future^30^. To evaluate NSUN2 activity, we incubated 200 nM NSUN2 with 250 ng/µL tRNA and 1 µM SAM for varying durations (0–60 min) and measured the SAH content formed during the reaction using the split aptamer and MST-based evaluation (Fig. 4A, B). NSUN2, a Rossmann-fold MTase, is prone to thermal unfolding^31^. In the storage buffer (25 mM HEPES pH 7.5, 300 mM NaCl, 10% glycerol, 0.04% Triton-X100, 0.5 mM TCEP) at 37 °C, we found NSUN2 has a catalytic t_1/2_ of ~4 h highlighting its intrinsic instability. We aimed to establish buffer and temperature conditions that allow the maintenance of catalytic competency for at least 14 days. Because the literature suggested potential benefits from the addition of BSA on protein stability, we conducted a series of incubation experiments at 4 °C and 20 °C in storage buffer, with and without 0.5% BSA (w/w) (Fig. 4C, D)^32^. After the designated incubation period, we initiated enzymatic reactions with the appropriate tRNA substrate and SAM. Our findings revealed that, in contrast to the aforementioned expectations, only the buffer without BSA at 4 °C maintained NSUN2’s catalytic competence for over 14 days (green curve, Fig. 4D).

**Fig. 5:**
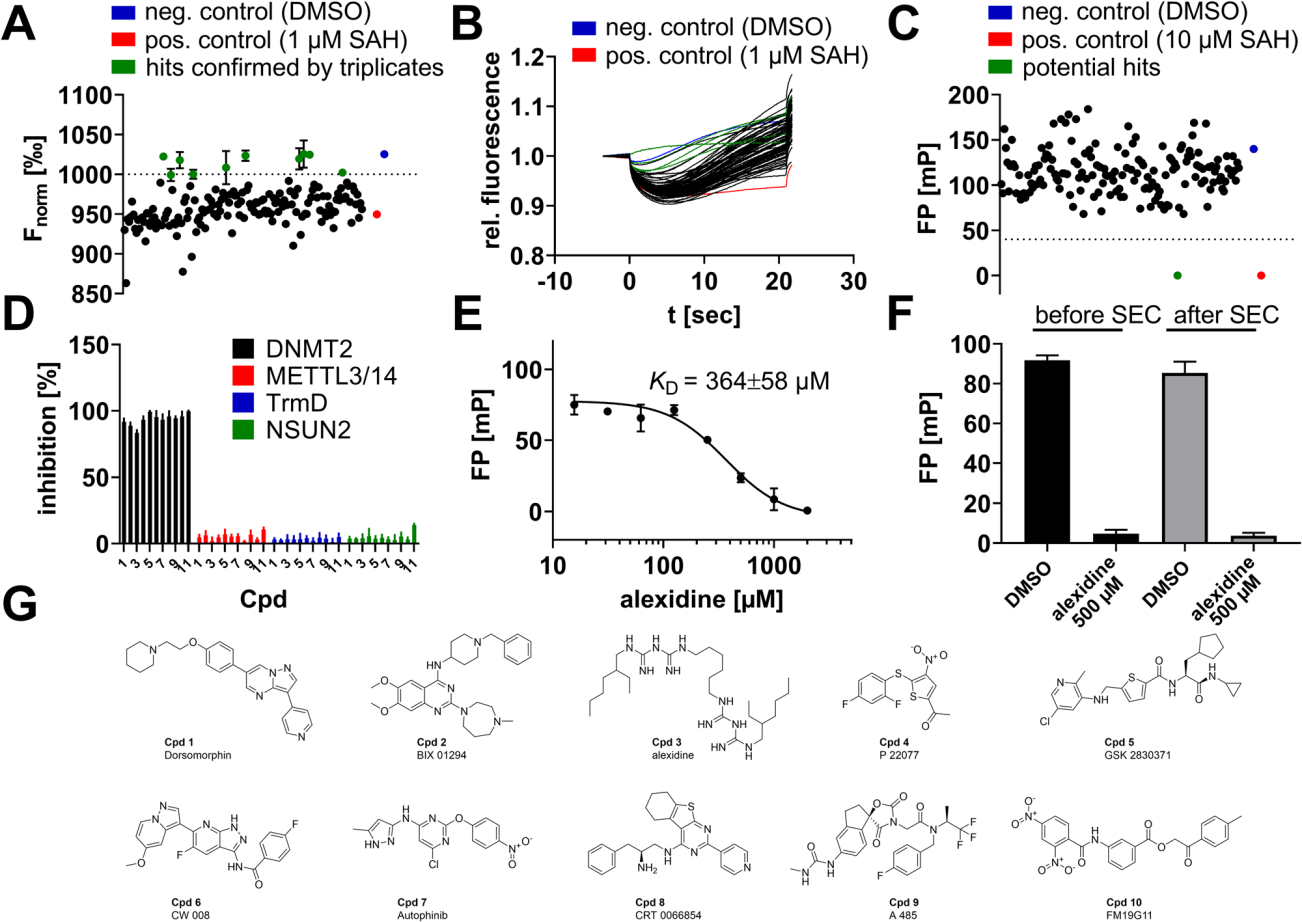
Pilot screening on DNMT2 using the epigenetic-focused compound library. **A** Screening a library including 160 drug-like compounds resulted in the identification of ten potential hit compounds (green) with an F_norm_ > 1000‰ (equals >80% inhibition). Initial hits were confirmed by triplicate validation (mean ± SD, *n* = 3). Positive reaction control: aptamer spiked with 1 µM SAH; negative control: aptamer mock-treated with DMSO. **B** Exemplary MST traces of the library’s first 80 compounds. **C** Orthogonal screening of the library by FP displacement experiments using FTAD as the fluorescent tracer. FP experiments reveal that only alexidine (**Cpd 3**, green) is binding to the SAH-binding site. **D** An inhibition selectivity panel was established by MST aptamer assays using the MTase assays described in this study and 500 µM of the respective hit compound. Only DNMT2 was inhibited significantly. **E** Alexidine’s (**Cpd 3**) dose-response curves and *K*_D_ determination by DNMT2 FP assays (mean ± SD, *n* = 3). **F** FP assays to confirm the reversibility of alexidine binding to DNMT2. The polarization of the DNMT2-FTAD complex (96 mP) is displaced in the presence of alexidine (5 mP) and can be effectively restored by analytical size-exclusion chromatography (92 mP). A second treatment with alexidine leads to a repeated FP displacement (4 mP). **G** Chemical structures of the identified DNMT2 hits.

**Fig. 6:**
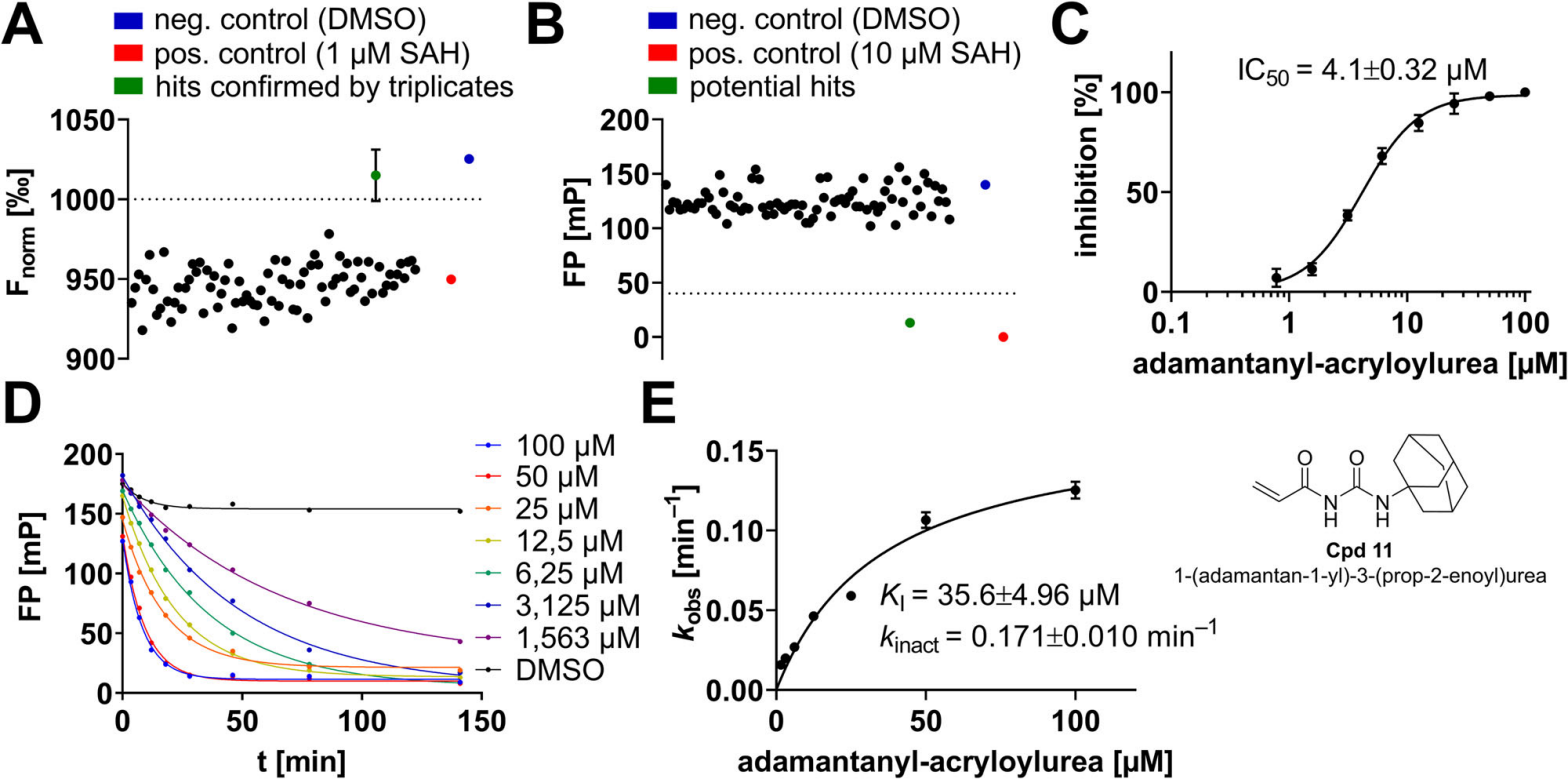
Pilot drug screening on DNMT2 using an in-house cysteine-focused covalent warhead library. **A** Screening a library including 80 cysteine-focused covalent compounds resulted in the identification of one single hit compound (green) with an F_norm_ > 1000‰ (equals >90% inhibition). Initial hits were confirmed by triplicate validation (mean ± SD, *n* = 3). Positive reaction control: aptamer spiked with 1 µM SAH; negative control: aptamer mock treated with DMSO. **B** Orthogonal screening of the library by fluorescence polarization displacement experiments using FTAD as the fluorescent tracer. FP experiments reveal that only adamantanyl-acryloylurea (**Cpd 11**, green) is binding to the SAH-binding site. **C** MST-derived dose-response curve for adamantanyl-acryloylurea (**Cpd 11**) including the chemical structure. All inhibitor characterizations were performed in triplicates (mean ± SD, *n* = 3). **D** FP assay with **Cpd 11** showing time-dependent DNMT2 inhibition with hyperbolic FP displacement plots. **E** Covalent dose-response analysis of subfigure D: *k*_obs_ vs. [I] for the determination of covalent inhibition constants (*K*_I_, *k*_inact_)^35,36^.

Finally, we utilized the newly identified MST assay for a pilot drug screening on the potential cancer drug target DNMT2, which introduces the m^5^C modifications to various tRNAs^24^. Using an enzymatic tRNA methyltransferase assay screening setup, we have tested a commercial epigenetic target-focused library including 160 drug-like compounds (Tocris) and an in-house covalent cysteine-focused warhead screening library (80 compounds) to identify novel DNMT2 binding scaffolds suitable for subsequent lead optimization (Figs. 5, 6). The drug candidate library was added to a master mix of recombinantly expressed DNMT2 protein (250 nM) with 5 µM tRNA and 0.9 µM SAM. The final drug compound concentration was adjusted to 500 µM for the epigenetic drug library (Fig. 5) resp. 100 µM for the covalent compound library (Fig. 6), and the enzymatic reaction was incubated for 30 min at 25 °C. Subsequently, enzymatic reactions were terminated by the addition of 0.3% SDS, and the product SAH was quantified by the split aptamer MST assay (Figs. 5A, B, 6A). Compounds that displayed F_norm_ values > 1000‰ were considered primary screening hits (equals >80% inhibition).

By this, we identified ten potential non-covalent hit compounds (Fig. 5G). Among those hits, alexidine (**Cpd 3**) was found to be the most promising hit as this was the only compound that showed inhibition at concentrations <500 µM and was able to compete with the fluorescent SAH-analog (5-FAM-triazolyl-adenosyl-diaminobutyric acid; FTAD) tracer during fluorescence polarization (FP) displacement experiments (Fig. 5C, E; *K*_D_ = 364 µM)^28^. Of note, the other nine compounds (Fig. 5G) were not able to displace the FTAD tracer during FP experiments, nor did they inhibit DNMT2 at decreased compound concentrations (200 µM) using the enzymatic aptamer assay.

We speculated these hits might bind very weakly to the tRNA-DNMT2 interaction site and by this inhibit DNMT2 activity. To test this hypothesis, we performed an electrophoretic mobility shift assay (EMSA) using tRNA^Asp^ (5 µM) and DNMT2 (10 µM) compared to the tRNA sample in absence of the DNMT2 protein (SI Fig. 4)^33^. Using this assay, the heterodimeric DNMT2-tRNA complex resulted in a significant electrophoretic shift compared to the native tRNA sample. Subsequently, we treated the DNMT2-tRNA complex with all hit compounds at 500 µM and observed their potential to disrupt the complex. Interestingly, six out of ten compounds showed the potential to reverse the complex formation (namely **Cpds 1, 2, 5, 6, 8**, and **9**) providing first-in-class (yet low affinity) starting points for DNMT2-tRNA interaction inhibitors. Of note, **Cpd 3** (alexidine) did not influence DNMT2-tRNA complex formation indicating its selective binding to the SAH binding pocket, and also, **Cpds 4, 7**, and **10** were found unable to disrupt the protein-tRNA complex, rendering these three compounds as DNMT2 inhibitors with an unknown mode of action.

Next, we investigated the target selectivity of all hit compounds by inhibition experiments using the MTases characterized in this study (DNMT2, METTL3/14, TrmD, and NSUN2) as a selectivity panel. Of note, none of the identified hit compounds inhibited any other RNA MTases at ligand concentrations as high as 500 µM (Fig. 5D), rendering these compounds DNMT2 selective hits. While **Cpd3** (alexidine) could be identified as a DNMT2 inhibitor during MST- and FP- experiments, the ligand’s hydrophobic nature raised the concern this compound might be an aggregator of DNMT2. To exclude this possibility, we investigated the reversibility of alexidine binding using the FP displacement assay and separation of the alexidine ligand by small-scale size-exclusion chromatography (SEC)^34^. Here, we found that the decrease in FP (92 to 5 mP) by alexidine displacing the fluorescent tracer FTAD is almost quantitatively reversed after SEC removal of alexidine (Fig. 5F). Furthermore, the restored FP levels can be displaced again by alexidine, and hence, this compound is a fully reversible binder resp. inhibitor of DNMT2 SAH pocket.

Lastly, we used the DNMT2 aptamer assay for a pilot screening of a cysteine-focussed covalent inhibitor library, since DNMT2 Cys-79 was previously identified as a valid target for covalent inhibitor development^26^. We screened this in-house library (*N* = 80 compounds) analogously to the non-covalent compound library, but the final compound concentration was adjusted to 100 µM. By this, we identified one single hit compound **Cpd 11** (adamantanyl-acryloylurea) which inhibited DNMT2 > 90% at 100 µM (Fig. 6A), displaced the FP-probe FTAD effectively (Fig. 6B), and did not inhibit any of the other MTases (Fig. 5D). Dose-response experiments revealed a single-digit micromolar inhibition constant (Fig. 6C; IC_50_ = 4.1 µM) and time-dependent displacement of the FTAD FP-tracer implying irreversible covalent inactivation of DNMT2 (Fig. 6D, E).

To summarize this study, we successfully developed an MST-based split aptamer assay for detecting RNA MTase activity, offering improved sensitivity and cost-effectiveness over existing methods. The assay was validated across multiple MTases, demonstrating high reproducibility and potential for drug discovery and high-throughput screenings.

## Methods

### RNA aptamer

The following two RNA aptamer sequences were synthesized and HPLC-purified by GenScript on a 500 nmol and yielded 50 nmol of the final split aptamer oligonucleotides (total: $759; sufficient for 100k individual experiments in 10 µL MST capillaries; <$0.001/per assay reaction).

aptaSAH1: TAMRA-5′-CUGCCGAGGAGCGCUGCGACCCUUUAAUUCGGGGGCCAGGCUCGGCAAUGAUGCC-3′

aptaSAH2: 5′-AUGAUCAACGGCGCUCGC-3′-FAM

### Microscale thermophoresis MTase assay

MTase enzyme reaction mixtures were prepared with buffer and substrate conditions as previously described^8,10,29^. Hence, the following enzyme-specific assay buffers and methylation conditions were used:

DNMT2 & *S. aureus* TrmD: 100 mM Tris-HCl, pH 8, 100 mM NH_4_OAc, 0.1 mM EDTA, 10 mM MgCl_2_, 10 mM DTT. For TrmD: 50 nM TrmD, 2 µM SAM, 1 µM tRNA^Leu^. For DNMT2: 250 nM DNMT2, 900 nM SAM, 5 µM tRNA

METTL3/14: 20 mM Tris-HCl, pH 7.5, 20 mM KCl, 0.25 mM MgCl_2_, 1 mM DTT, 0.01% Triton-X100. 10 nM METTL3/14, 500 nM SAM, 120 nM RNA substrate.

NSUN2: 50 mM Tris-HCl, pH 7.5, 5 mM EDTA, 5 mM MgCl_2_, 1.5 mM DTT. 200 nM NSUN2, 1 µM SAM, 250 ng/µL tRNA (total from *E. coli*).

CAVE: It is important to use high-quality SAM for assay preparations since frequent contamination in commercial SAM is in fact SAH which shall be detected by this assay, and thus, can distort the final assay results. We found SAM from New England Biology (B9003S) to be sufficiently pure if adequately stored (–20 °C, aliquot but do not dilute!) and used within the shelf life given by the manufacturer. When in doubt about a SAM stock solution, we found the quality can be easily confirmed by LC/MS.

For MTase assay detection by MST, enzyme reaction mixtures with a total volume of 10 µL per single treatment condition were prepared and incubated for the desired reaction times. Inhibitors were added from corresponding DMSO stocks to a final DMSO concentration of max. 5%. Afterward, reactions were quenched at a given reaction time by the addition of 1 µL sodium dodecyl sulfate (SDS, solution, 3% in water) per 10 µL reaction mixture and mixed well by pipetting. Subsequently, 1 µL of a 10× aptamer-containing detection master mix (200 mM Tris, pH 8.5, 200 mM MgCl_2_, 3 M NaCl, 10 mM urea, 0.2% Brij, 500 nM aptaSAH1, 500 nM aptaSAH2) was added to the quenched MTase reaction and incubated for 1 h at room temperature. Reaction mixtures were loaded directly to MST capillaries without further purification or sample processing.

MST experiments were carried out in at least technical triplicates using a Monolith NT.115 instrument (NanoTemper Technologies) using Monolith standard capillaries and the blue excitation laser setup. The instrument was calibrated according to the manufacturer’s instructions. General settings were applied for all MST experiments as follows: manual temperature control: 25 °C, fluorescence measurement before MST: 4 s, MST (IR laser) on: 20 s, fluorescence after MST: 2 s, delay: 25 s. The laser power was adjusted to optimize the signal-to-noise ratio and the fluorescence signal. LED and MST power settings were chosen individually for each sample by adjusting the LED power to yield fluorescence signals of at least 300 units (blue laser) or 3000 units (red laser) and the MST power to achieve an appropriate thermophoretic response (standard setting: medium MST power). MST measurements were analyzed using the NT analysis software (version 1.5.41) and exported for statistical analysis and plotting in GraphPad Prism 7.01.

### FRET-based MTase assay

MTase reaction mixtures were prepared analogously to the MST-based detection approach but on a 30 µL scale (minimum volume for 96-well half-area plates). Reactions were quenched at a given time by 0.3% SDS and supplemented with 3 µL of the 10× aptamer-containing detection master mix. Reaction mixtures were transferred in technical triplicates to black Greiner 96-well half-area plate and incubated for 1 h at room temperature. Afterward, plates were measured using a Tecan Spark 10 M plate reader equipped with a monochromator setup (λ_ex_ = 485 nm; λ_em_ = 600 nm). Statistical analysis and plotting were performed in GraphPad Prism 7.01.

### Orthogonal MTase enzyme assays

Radiometric DNMT2 assays were carried out as described previously^26^. HTRF MTase assays with the commercial AptaFluor SAH assay kit (BellBrook Labs) were performed as described in the manufacturer’s manual in 96-well format^18^.

The inhibitors used in this study include **SHO108** (DNMT2 inhibitor, synthesized in-house, (*S*)-2-amino-4-((((2 *R*,3*S*,4 *R*,5 *R*)-5-(6-amino-9*H*-purin-9-yl)-3,4-dihydroxytetrahydrofuran-2-yl)methyl)(prop-2-yn-1-yl)amino)butanoic acid), **SH112** (DNMT2 inhibitor, synthesized in house, (*S*)-2-amino-4-((((2 *R*,3*S*,4 *R*,5 *R*)-5-(6-amino-9*H*-purin-9-yl)-3,4-dihydroxytetrahydrofuran-2-yl)methyl)(4-chloro-3-(trifluoromethyl)benzyl)amino)butanoic acid), **STM2457** (METTL3/14 inhibitor, commercial, biomol) and 5-phenylthieno[2,3-*d*]pyrimidin-4(1*H*)-one (*S. aureus* TrmD inhibitor, commercial, BLDpharm)^7,8,10^. A compound library for the DNMT2 pilot screening (including **Cpd 1–10**) was commercially obtained (Tocriscreen Epigenetics 3.0, Cat. No. 7578). **Cpd 11** (adamantanyl-acryloylurea) was obtained commercially from Enamine. *K*_I_ values were calculated by correcting IC_50_ values to the zero-substrate concentration by the Cheng-Prusoff equation: *K*_I_ = IC_50_/(1 + ([S]/*K*_*M*_)).

### Fluorescent tracer displacement assay (FTAD-MST assay)

Fluorescent tracer displacement MST assays for orthogonal ligand evaluation were conducted as described previously^27^. The samples contained 2 µM DNMT2, 100 nM FTAD probe, and 1.3% DMSO in MST buffer (50 mM HEPES, 150 mM NaCl, 1 mM dithiothreitol (DTT), 0.1% PEG-8000, 0.05% polysorbate-20, pH 7.4) supplemented with variable concentrations of the screening compounds. All samples were measured in triplicates in Monolith standard capillaries at 25 °C. The acquisition mode was set to “Nano-Blue” with an excitation power of 30% and a medium MST power. MST measurements were analyzed using the NT analysis software (version 1.5.41) and exported for statistical analysis and plotting in GraphPad Prism 7.01.

### Fluorescence polarization (FP-assay)

Fluorescence polarization assays were conducted with a similar sample composition as described for FTAD-MST assays as described previously^28^. Triplicates (50 µL) were prepared in black 96-well half-area plates (Greiner) and measured using a Spark 10 M plate reader (Tecan) equipped with polarization filters coupled to a monochromator setup (FTAD: λ_ex_ = 480 nm, λ_em_ = 530 nm). Polarization values (in mP) were calculated from polarization-specific parallel and orthogonal fluorescence intensities according to the Tecan in-built calculation routine.

Reversibility of alexidine binding to DNMT2 was evaluated with a modified FP-assay setup. Briefly, reaction mixtures containing alexidine were prepared in black 96-well half-area plates, and after FP measurement, the volume of one well (50 µL) was collected and applied to a Zeba™ Spin Desalting Column (7 K MWCO) while 5 µL of a 10x DNMT2 buffer solution (500 mM HEPES, 1.5 M NaCl, 10 mM dithiothreitol (DTT), 1% PEG-8000, 0.5% polysorbate-20, pH 7.4) was placed in the collection tube attached to the SEC column. This procedure separated alexidine and restored the initial composition of the DNMT2 assay solution.

Kinetic FP assays were performed to assess the time-dependent inactivation of DNMT2 treated with covalent inhibitors. Briefly, samples contained 2 µM DNMT2, 100 nM FTAD probe, and 1.3% DMSO in DNMT2 MST buffer. Inhibitors were added from DMSO stocks. Negative inhibition control was performed by mock treatment with DMSO. Reactions were initiated by the addition of the **Cpd 11** (adamantanyl-acryloylurea) and monitored for 120 min at 25 °C in a Tecan Spark 10 M plate reader (FTAD: λ_ex_ = 480 nm, λ_em_ = 530 nm) The irreversible inhibition kinetics were analyzed as described previously^35,36^.

### Electrophoretic mobility shift assay (EMSA)

DNMT2 (10 µM) was mixed with tRNA^Asp^ (5 µM) and treated with 500 µM of the respective screening hit compound in DNMT2 assay buffer (100 mM Tris-HCl, pH 8, 100 mM NH_4_OAc, 0.1 mM EDTA, 10 mM MgCl_2_, 10 mM DTT). Subsequently, reaction samples (each 5 µL) were loaded to a native 10% polyacrylamide gel and separated electrophoretically (1 h, 180 V) in 1x TBE. RNA was stained in situ with 1x GelRed and PAGE analysis was conducted by fluorescence scanning at an excitation wavelength of 532 nm (Amersham Typhoon 9400). Positive control: DNMT2+tRNA (band at high molecular weights); negative control: only tRNA (band at low molecular weights).

### Recombinant protein and RNA production

DNMT2, NSUN2, and *S. aureus* TrmD were recombinantly expressed and purified as described previously^8,37^. For NSUN2, instead of the previously described SEC buffer, a buffer containing 25 mM HEPES (pH 7.5), 300 mM NaCl, 10% Glycerol, 0.04% Triton X-100, and 0.5 mM TCEP was used for size-exclusion chromatography. Recombinant METTL13/14 was obtained commercially (BPS Bioscience, BPS-100105). tRNA substrates for DNMT2 and NSUN2 were prepared as described previously^8^. RNA substrates for TrmD (synthetic tRNA^Leu^: 5′-GCGAAGGUGGCGGAAUUGGUAGACGCGCUAGCUUCAGG UGUUAGUGUCCUUACGGACGUGGGGGUUCAAGUCCCCCCCCUCGCACCA-3′) and METTL3/14 (5′-AACUUAAUGUUGCAUUGGACUUGAGUUA-3′) were obtained commercially (GenScript).

### Reporting summary

Further information on research design is available in the Nature Portfolio Reporting Summary linked to this article.

## Supplementary information

Supplementary Information

Reporting Summary

## Data Availability

Authors can confirm that all relevant data are included in the paper and/or its supplementary information files.
