# Supplementary material for: A microscale thermophoresis-based enzymatic RNA methyltransferase assay enables the discovery of DNMT2 inhibitors
Source: Commun Chem. 2025 Feb 3;8:32. doi: 10.1038/s42004-025-01439-9 (PMC11790956; doi:10.1038/s42004-025-01439-9)

A Microscale Thermophoresis-based Enzymatic RNA Methyltransferase Assay Enables the Discovery of DNMT2 Inhibitors

Zarina Nidoieva[1, †], Mark O. Sabin[1, †], Tristan Dewald[1], Annabelle C. Weldert[1], Sabrina N. Hoba[1], Mark Helm[1], Fabian Barthels[1,2*]

†Authors have contributed equally

[1]Institute of Pharmaceutical and Biomedical Sciences, Johannes Gutenberg-University Mainz, Germany.

[2]Department of Molecular, Cellular, and Developmental Biology, Yale University, New Haven, Connecticut, USA.

*** Correspondence:**
Fabian Barthels
barthels@uni-mainz.de

Supplementary results

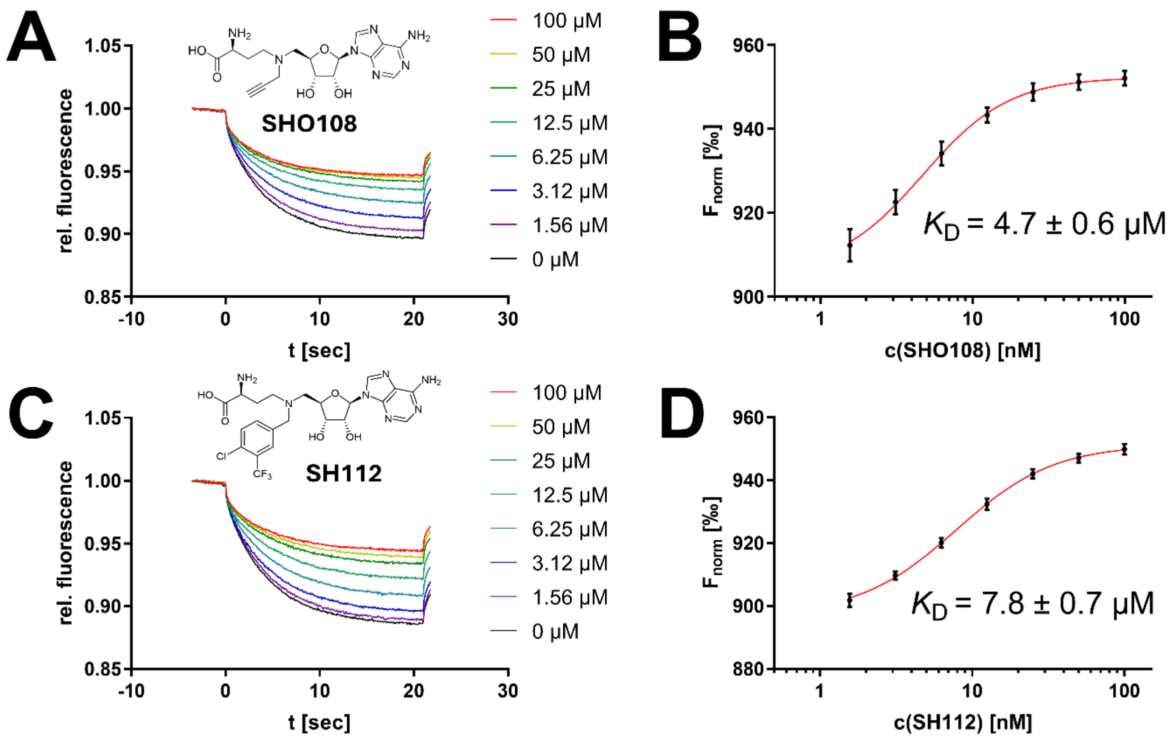

SI Figure 1. **Displacement of FTAD by literature-known inhibitors in a microscale thermophoresis displacement assay.** Data is given as mean ± SD of triplicates. (**A**) MST traces of SHO108. (**B**) MST-derived dose-response curves of SHO108. (**C**) MST traces of SH112. (**D**) MST-derived dose-response curves of SHO112

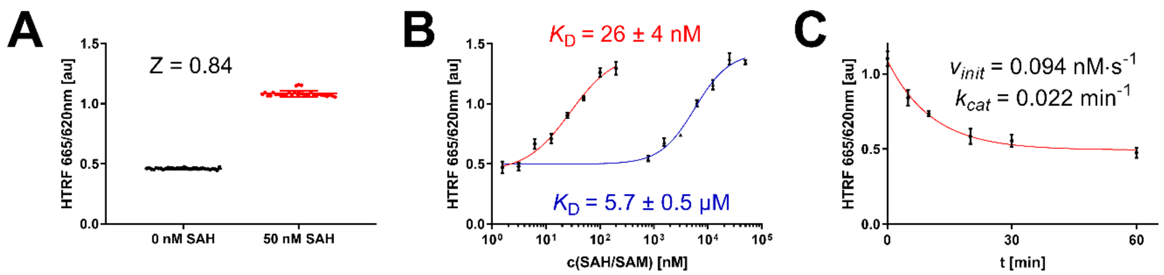

SI Figure 2. **Benchmarking of commercial AptaFluor SAH assay kit for comparison with the split aptamer described in this manuscript.** (**A**) Z-factor determination at 50 nM SAH (n=32) to evaluate the robustness and HTS-suitability of the commercial AptaFluor assay. (**B**) Dose-response curves of the AptaFluor assay in the presence of the SAH or SAM ligand by plate reader-based HTRF assays (λ_{ex}=332 nm; λ_{em}=620/665 nm). (**C**) Substrate conversion plots of the DNMT2 enzyme reaction (250 nM DNMT2, 5 µM tRNA, 0.9 µM SAM) incubated for variable durations (0–120 min) and yielded HTRF shifts for the determination of steady-state DNMT2 kinetics. Data is given as mean ± SD of triplicates.

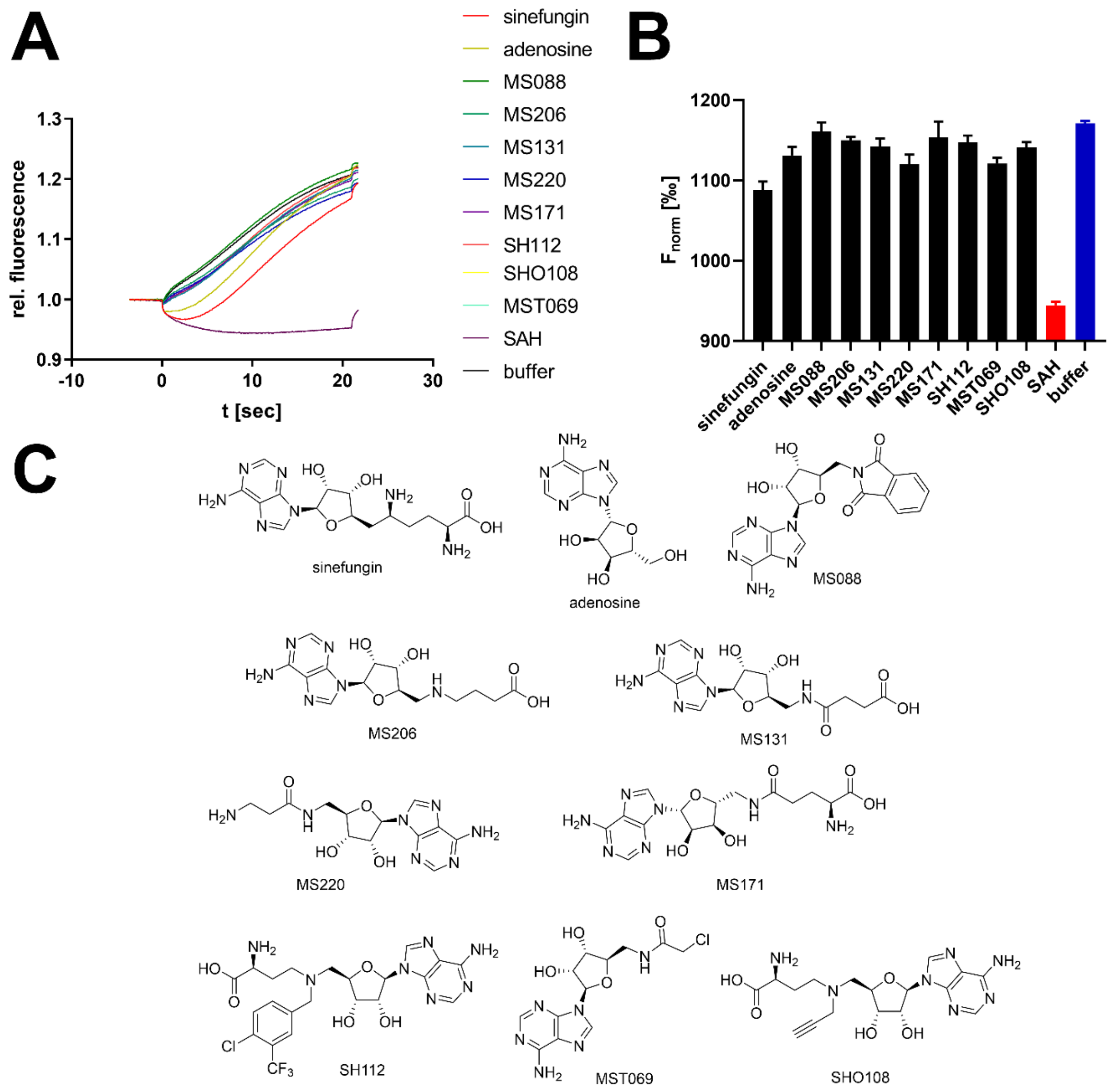

SI Figure 3. **SAH analog binding selectivity assessment of the SAH split aptamer by MST experiments.** (**A,B**) MST traces and shifts of aptaSAH1&2 (both 50 nM) in the presence of various SAH analogs (10 µM) indicated these compounds are not binding to the aptamer at relevant concentrations. Data is given as mean ± SD of triplicates. (**C**) Chemical structures of the investigated SAH analogs

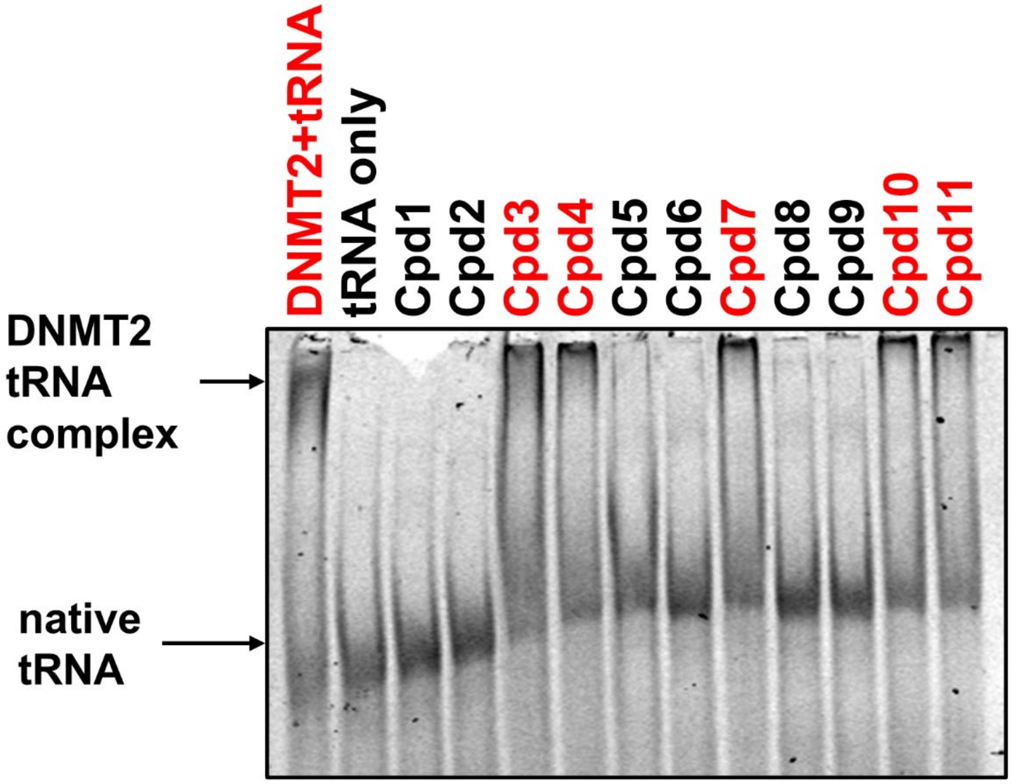

SI Figure 4. **Electrophoretic mobility shift assay (EMSA) to investigate the influence of screening hits on the DNMT2-tRNA complex formation.** DNMT2 (10 µM) was mixed with $tRNA^{Asp}$ (5 µM) and treated with 500 µM of the respective screening hit compound in DNMT2 assay buffer. All reaction samples (each 5 µL) were loaded to a native 10% polyacrylamide gel and separated electrophoretically (1 h, 180 V) in 1x TBE. RNA was stained in situ with 1x GelRed and PAGE analysis was conducted by fluorescence scanning at an excitation wavelength of 532 nm (Amersham Typhoon 9400). Positive control: DNMT2+tRNA (band at high molecular weights); negative control: only tRNA (band at low molecular weights). **Cpds 3, 4, 7, 10,** and **11** do not influence the complex formation between DNMT2 and tRNA, while **Cpds 1, 2, 5, 6, 8,** and **9** lead to a quantitative disruption of the DNMT2-tRNA complex.

Compound characterization

SHO108

^{1}H NMR (300 MHz, CD_3OD): δ / ppm = 8.23 (s, 1H), 8.06 (s, 1H), 5.83 (d, J = 4.9 Hz, 1H), 4.45 (t, J = 4.9 Hz, 1H), 4.20 (d, J = 7.0 Hz, 1H), 4.09 (t, J = 4.9 Hz, 1H), 3.88 (t, J = 2.8 Hz, 2H), 3.85–3.76 (m, 1H), 3.51–3.28 (m, 2H), 3.24 (t, J = 7.1 Hz, 2H), 2.96 (s, 1H), 2.22–2.04 (m, 1H), 1.99–1.84 (m, 1H). ^{13}C NMR (75.5 MHz, CD_3OD): δ / ppm = 172.2, 152.3, 149.7, 145.8, 144.5, 120.6, 91.1, 81.1, 80.7, 74.9, 73.4, 72.9, 56.8, 52.8, 52.5, 42.9, 26.3. FT-IR: ν / cm^{-1} = 3097, 1671, 1509, 1428, 1323, 1195, 1137, 837, 799, 723. $[\alpha]_D^{20}$ = +12 ° (10 mg/mL; MeOH). mp: 76–79 °C. ESI-MS: m/z calculated for $C_{17}H_{24}N_7O_5$ $[M+H]^+$ = 406.2 (100.0%), 407.2 (18.4%), 408.2 (1.6%), found: $[M+H]^+$ = 406.2 (100.0%), 407.2 (21.0%), 408.1 (2.5%). Purity: 97% (HPLC, 254 nm, MeCN/H_2O + 0.1% HCOOH = 20:80, t_R = 2.70 min).

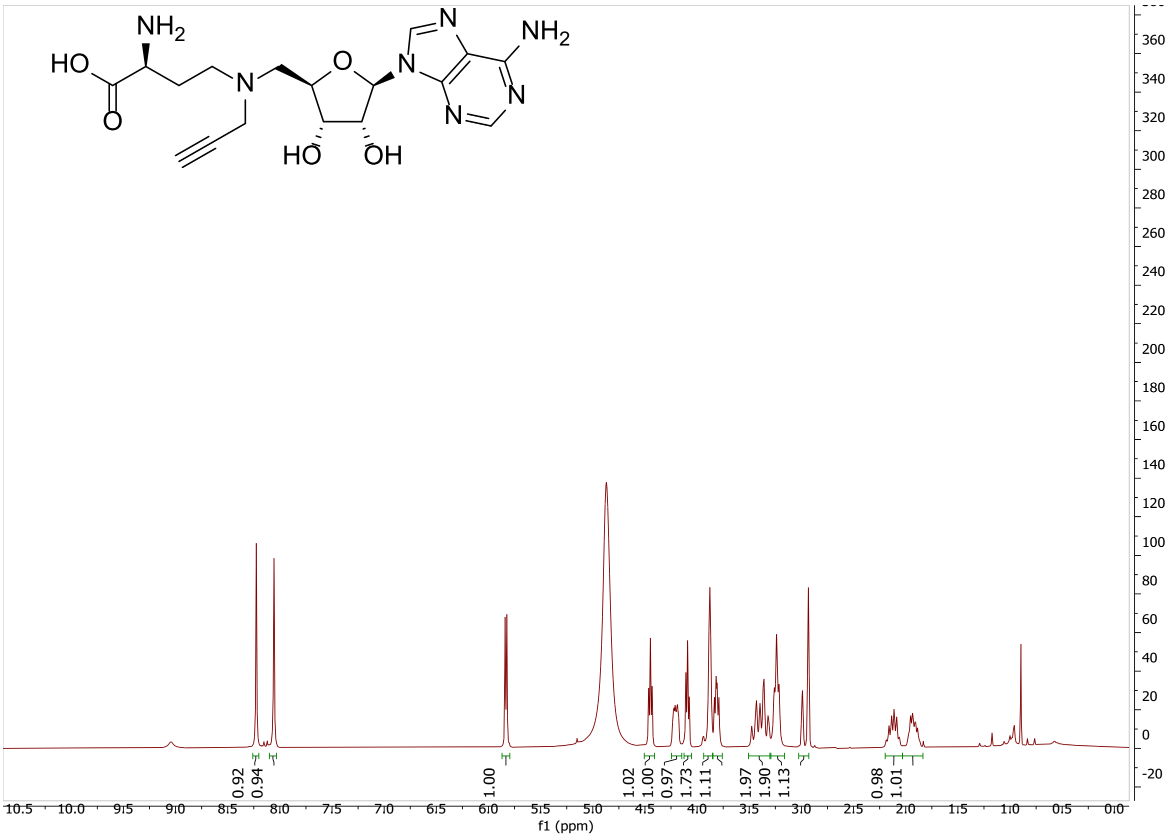

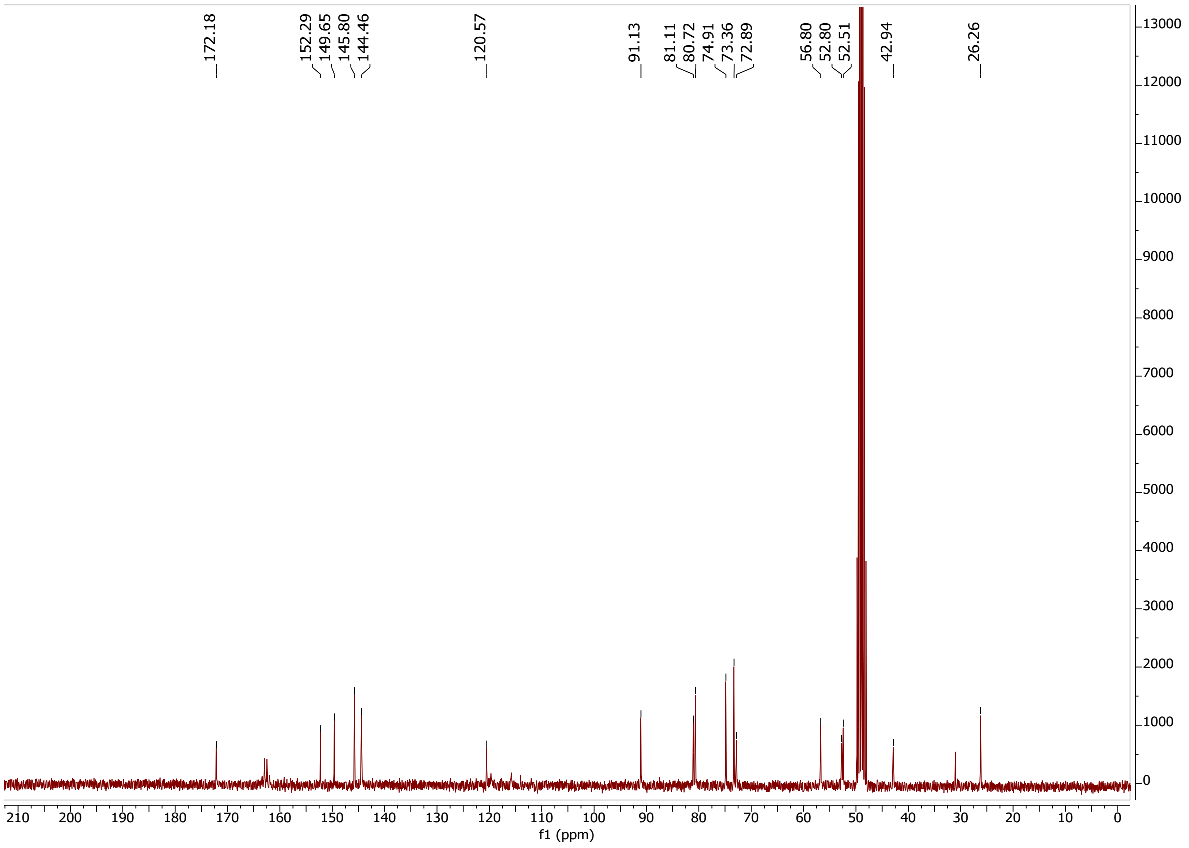
172.18
152.29
149.65
145.80
144.46
120.57
91.13
81.11
80.72
74.91
73.36
72.89
56.80
52.80
52.51
42.94
26.26
f1 (ppm)

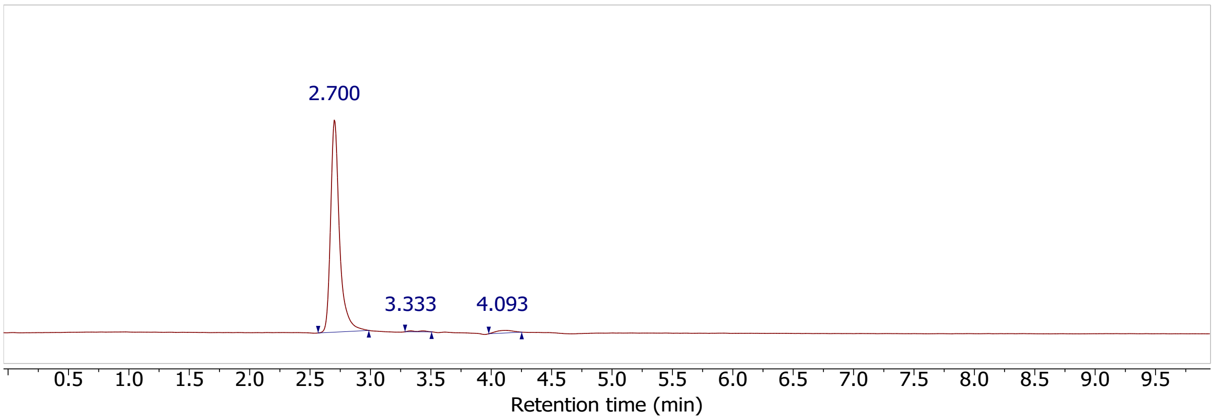
2.700
3.333
4.093
Retention time (min)

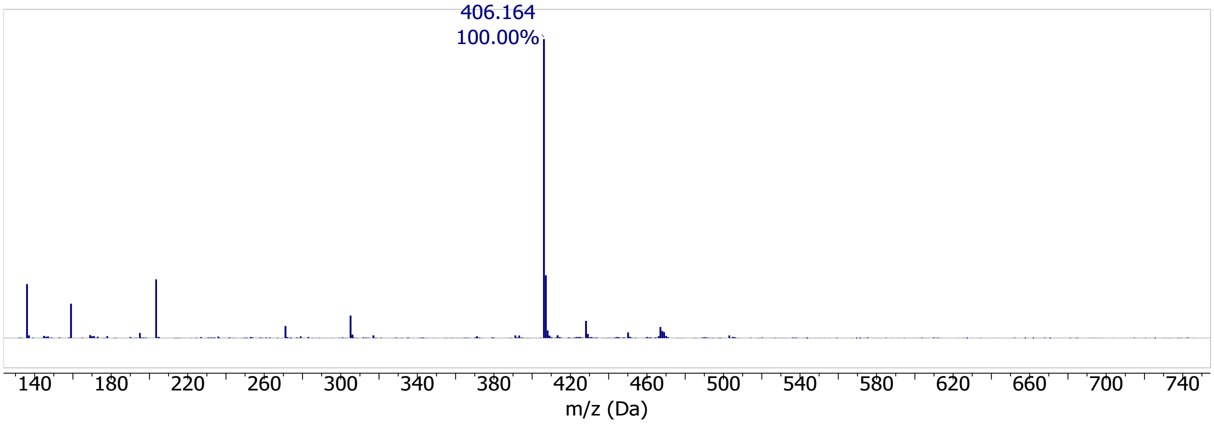
406.164
100.00%
m/z (Da)

SH112

^{1}H NMR (300 MHz, CD_3OD): δ / ppm = 8.20 (s, 1H), 8.10 (s, 1H), 7.67 (d, J = 2.1 Hz, 1H), 7.48 (dd, J = 8.2, 2.1 Hz, 1H), 7.32 (d, J = 8.2 Hz, 1H), 5.91 (d, J = 3.0 Hz, 1H), 4.37 (dd, J = 4.9, 3.0 Hz, 1H), 4.32–4.12 (m, 4H), 3.79 (dd, J = 7.9, 4.9 Hz, 1H), 3.43–3.18 (m, 4H, H-5), 2.28–2.12 (m, 1H), 2.11–1.95 (m, 1H). ^{13}C NMR (75.5 MHz, CD_3OD): δ / ppm = 171.9, 152.7, 149.5, 146.2, 144.3, 137.0, 134.3, 133.2, 132.1, 131.1, 129.6, 123.89, 121.0, 92.3, 80.2, 74.9, 73.4, 57.9, 56.0, 52.9, 52.7, 26.6. FT-IR: ν / cm^{-1} = 3080, 1663, 1506, 1484, 1427, 1322, 1267, 1179, 1129, 1039, 975, 834, 799, 722, 664. $[\alpha]_D^{20}$ = +15 °(10 mg/mL; MeOH); mp: 79–82 °C; ESI-MS: m/z calculated for $C_{22}H_{25}ClF_3N_7O_5$ $[M+H]^+$ = 560.2 (100.0%), 561.2 (23.8%), 562.2 (32.0%), found: $[M+H]^+$ = 560.1 (100.0%), 561.1 (26.4%), 562.1 (35.9%),. Purity: 96% (HPLC, 254 nm, MeCN/H_2O + 0.1% HCOOH = 20:80, t_R = 3.42 min).

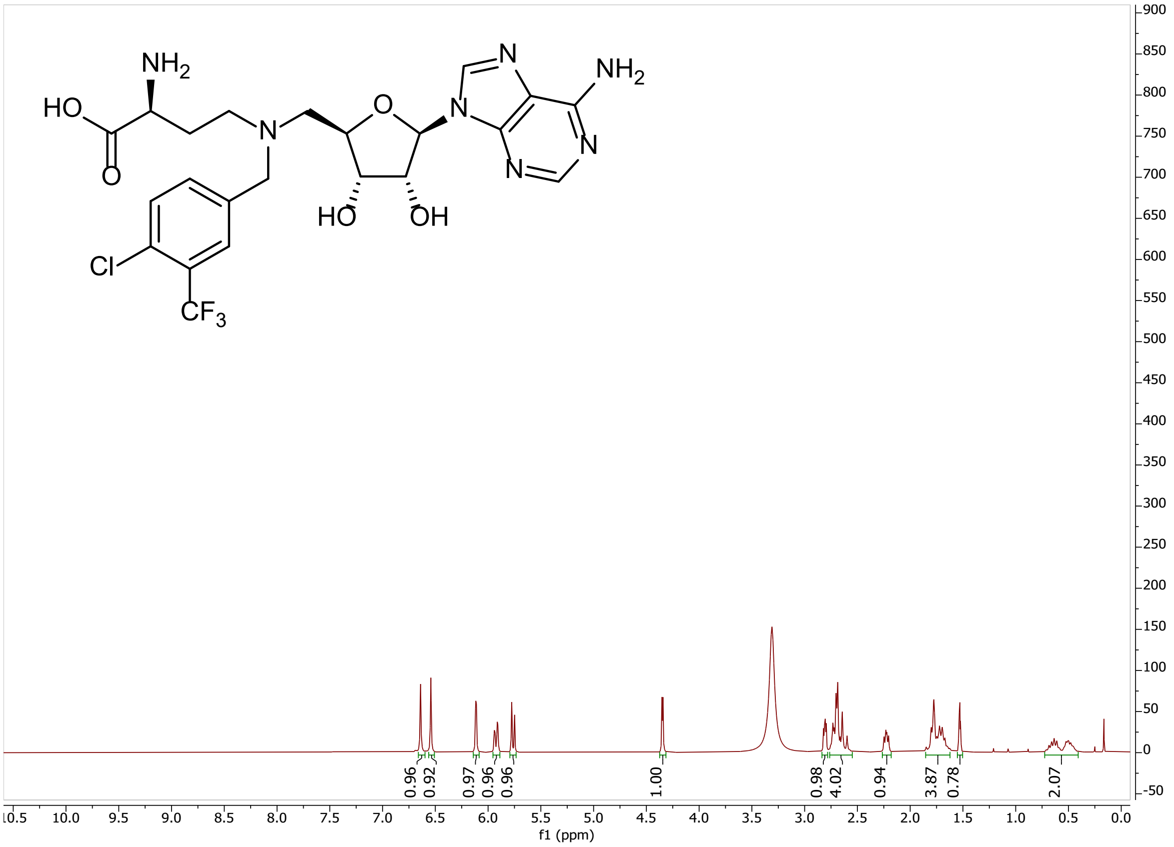

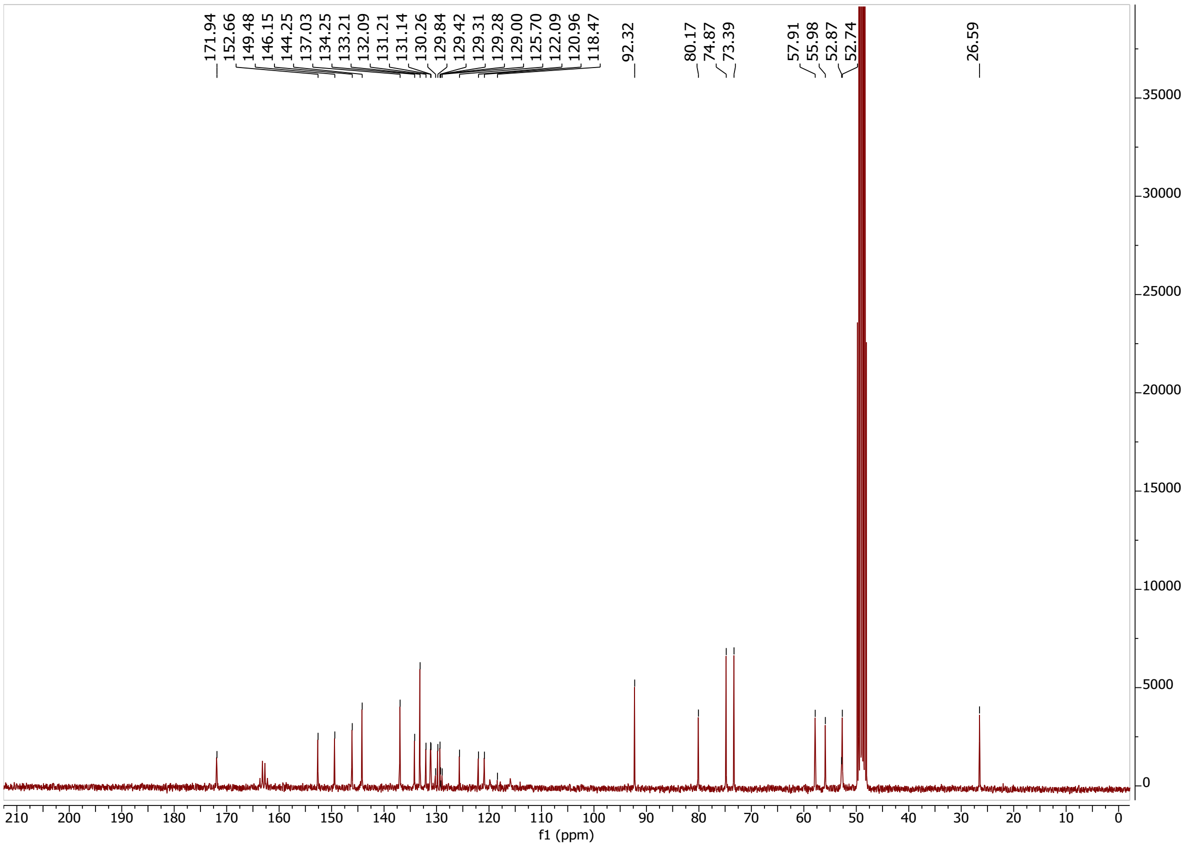
171.94
152.66
149.48
146.15
144.25
137.03
134.25
133.21
132.09
131.21
131.14
130.26
129.84
129.42
129.31
129.28
129.00
125.70
122.09
120.96
118.47
92.32
80.17
74.87
73.39
57.91
55.98
52.87
52.74
26.59
35000
30000
25000
20000
15000
10000
5000
0
210 200 190 180 170 160 150 140 130 120 110 100 90 80 70 60 50 40 30 20 10 0
f1 (ppm)

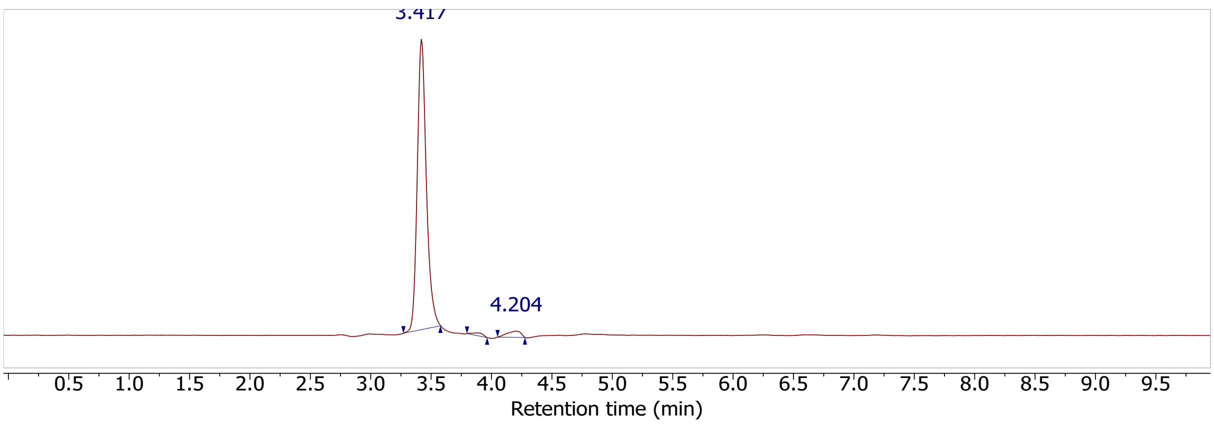
3.417
4.204
0.5 1.0 1.5 2.0 2.5 3.0 3.5 4.0 4.5 5.0 5.5 6.0 6.5 7.0 7.5 8.0 8.5 9.0 9.5
Retention time (min)

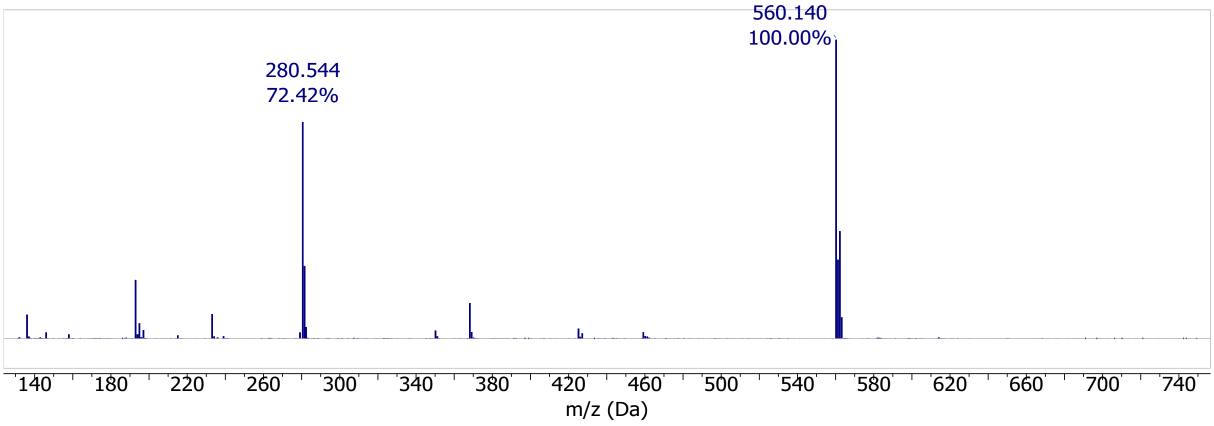
560.140
100.00%
280.544
72.42%
140 180 220 260 300 340 380 420 460 500 540 580 620 660 700 740
m/z (Da)

5-phenylthieno[2,3-*d*]pyrimidin-4(1*H*)-one

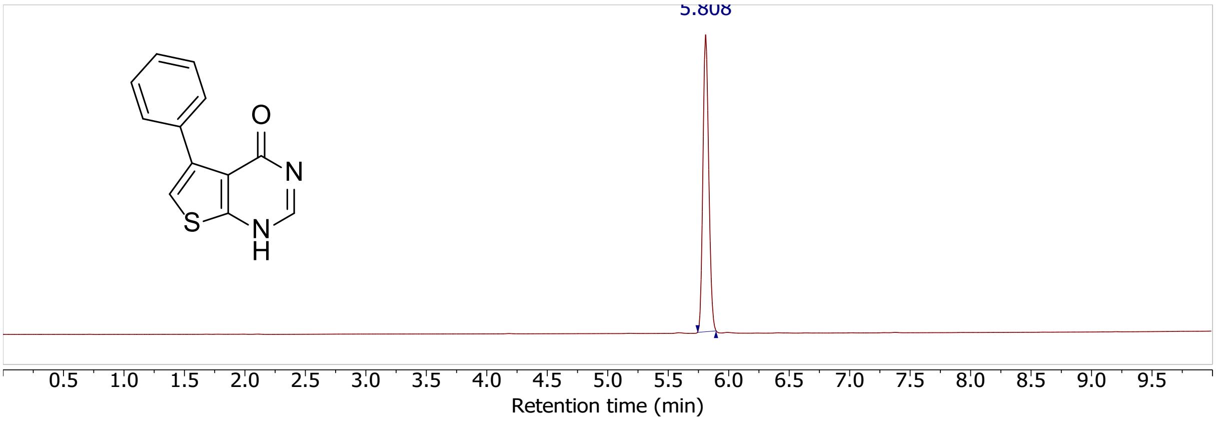

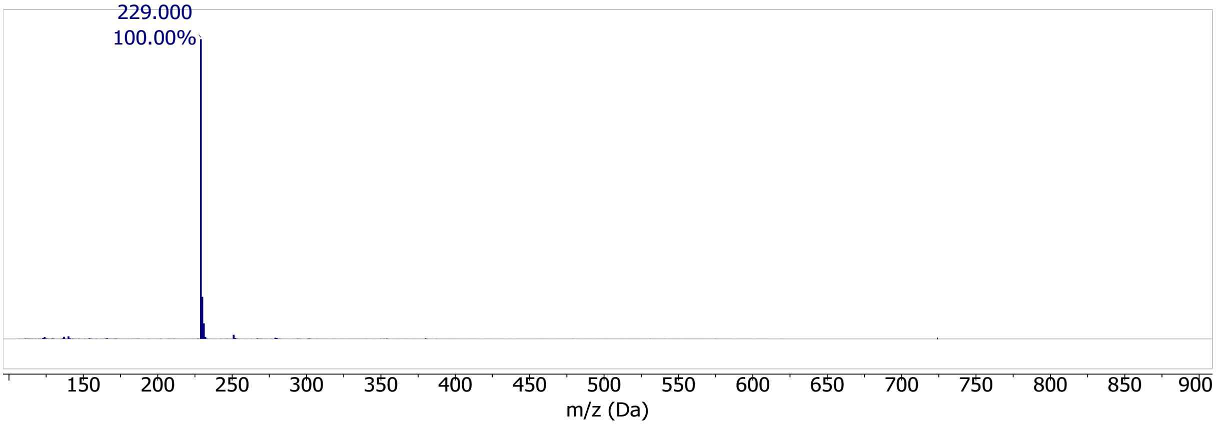

STM2457

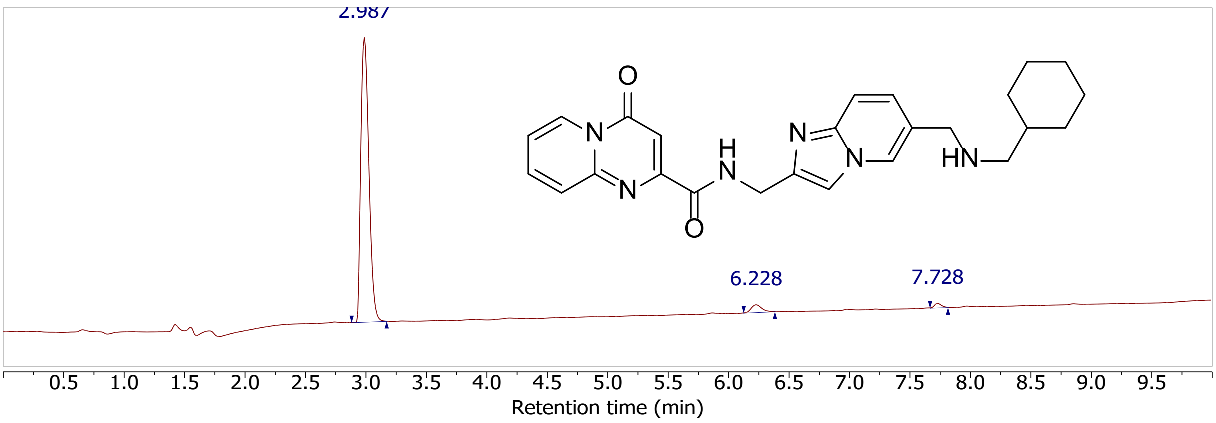

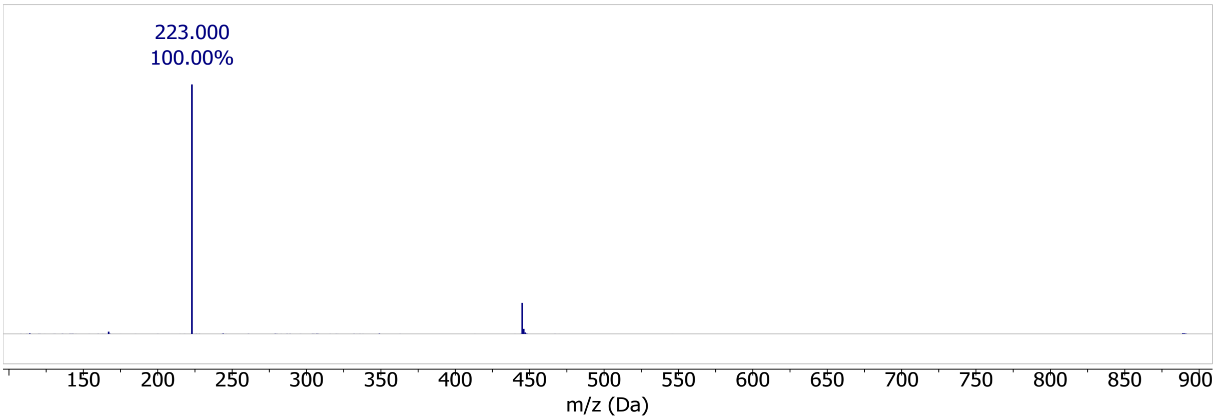

Cpd 1 (1C8, Dorsomorphine)

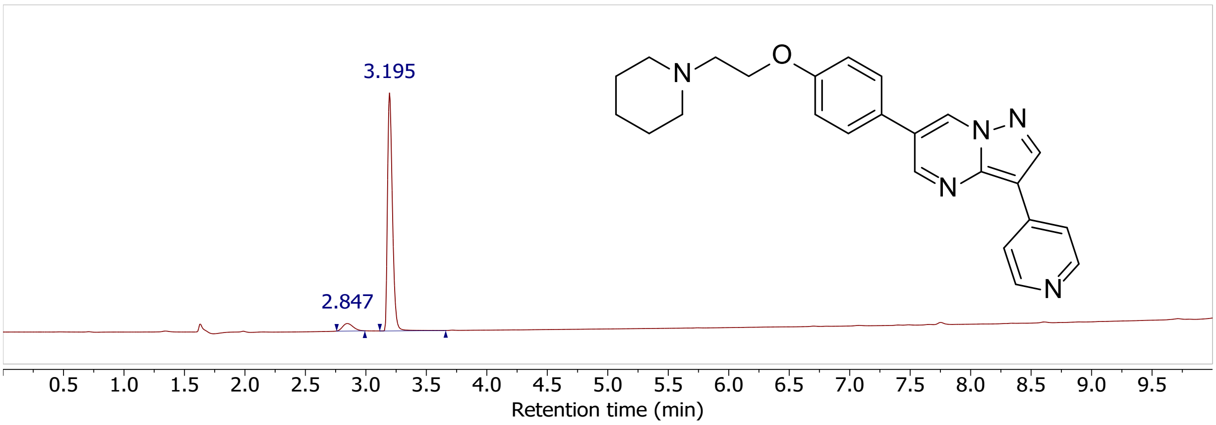

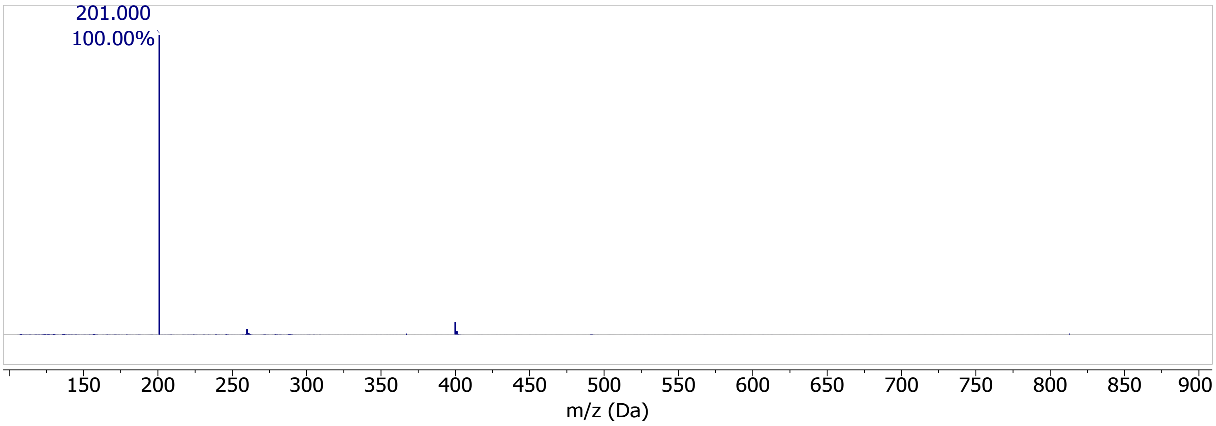

Cpd 2 (1D3, BIX01294)

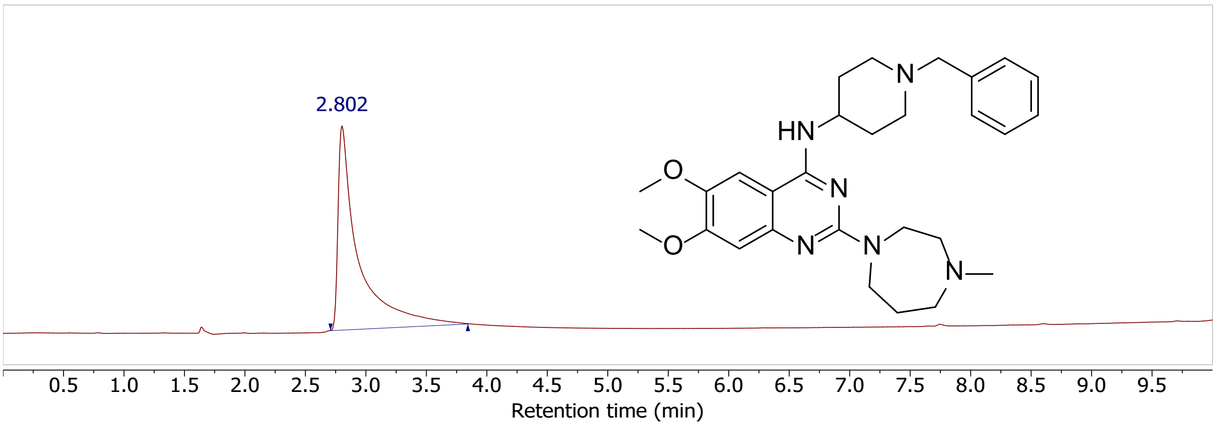

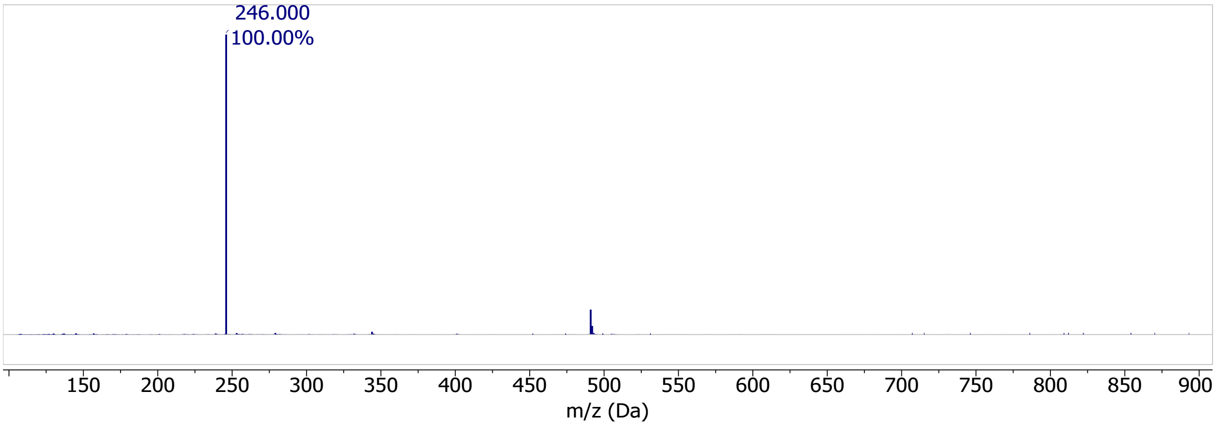

Cpd 3 (1D9, Alexidine)

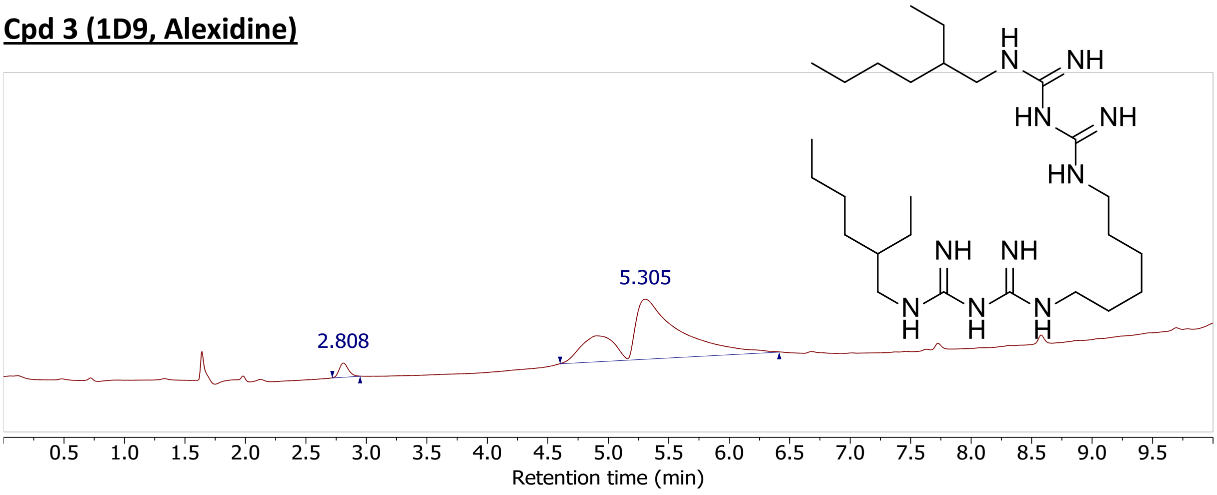

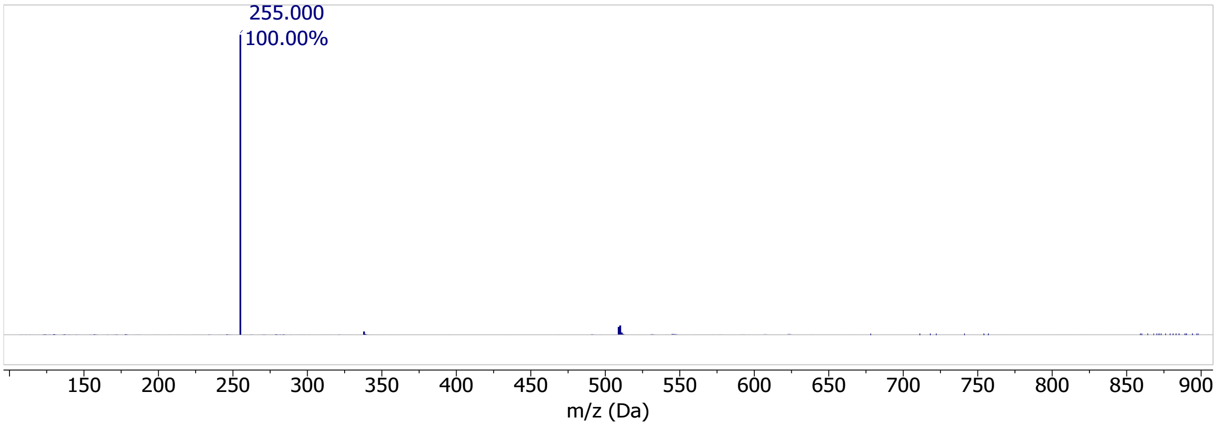

Cpd 4 (1E8, P22077)

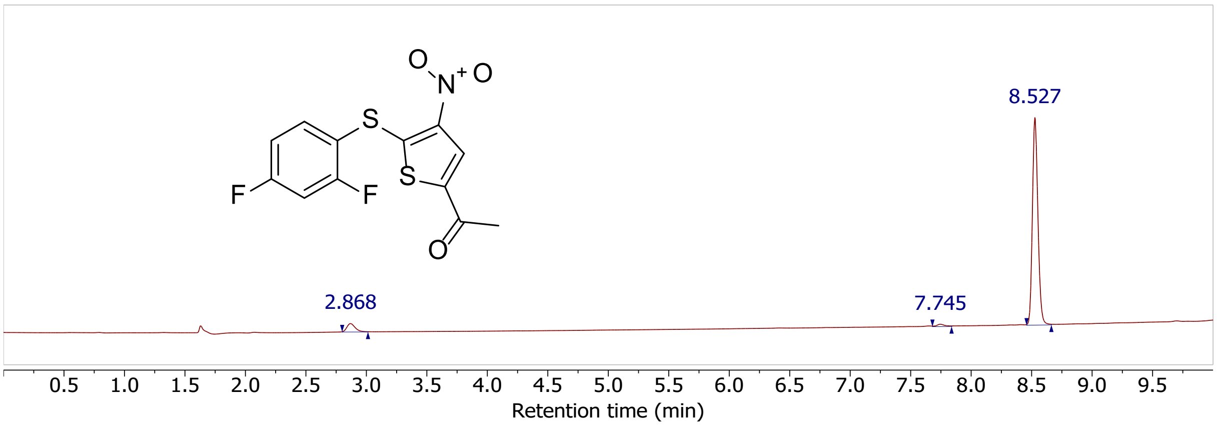

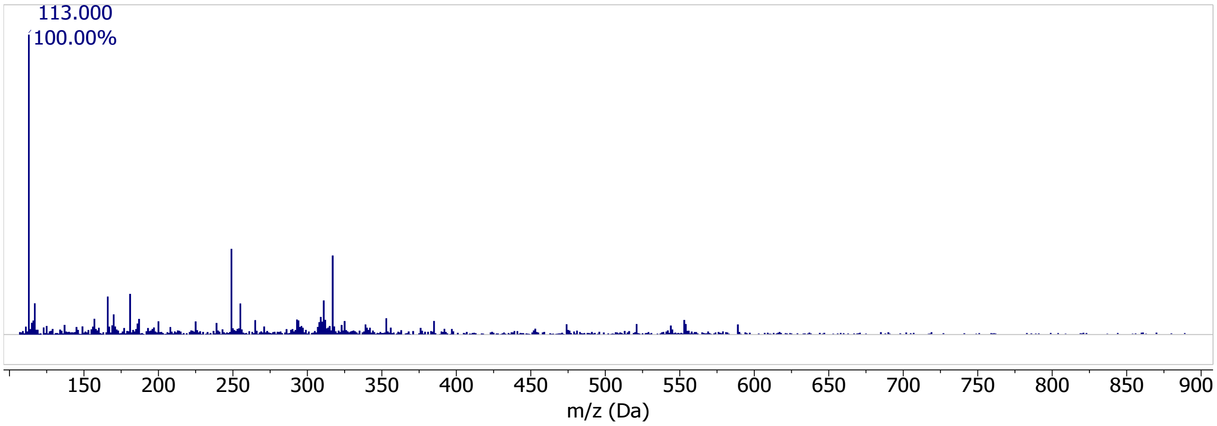

Cpd 5 (1G10, GSK 2830371)

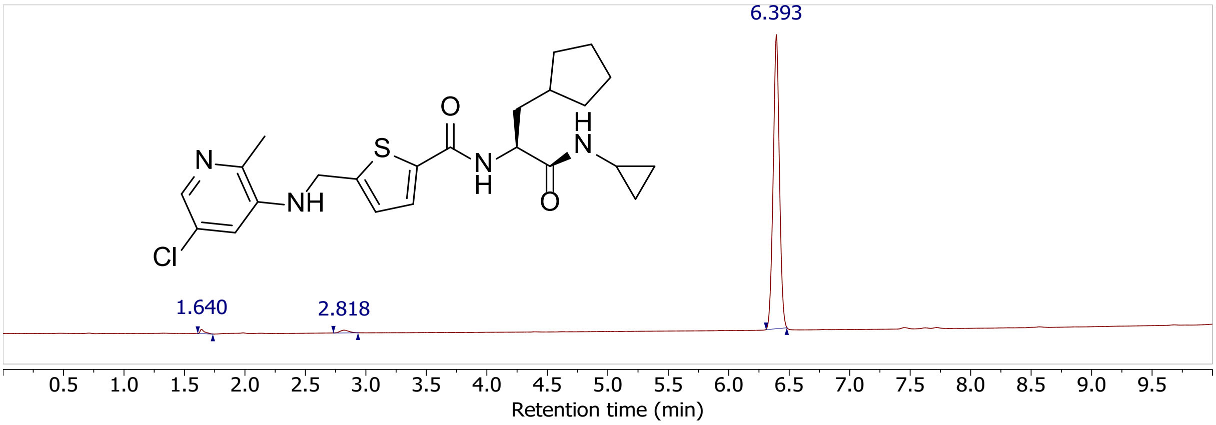

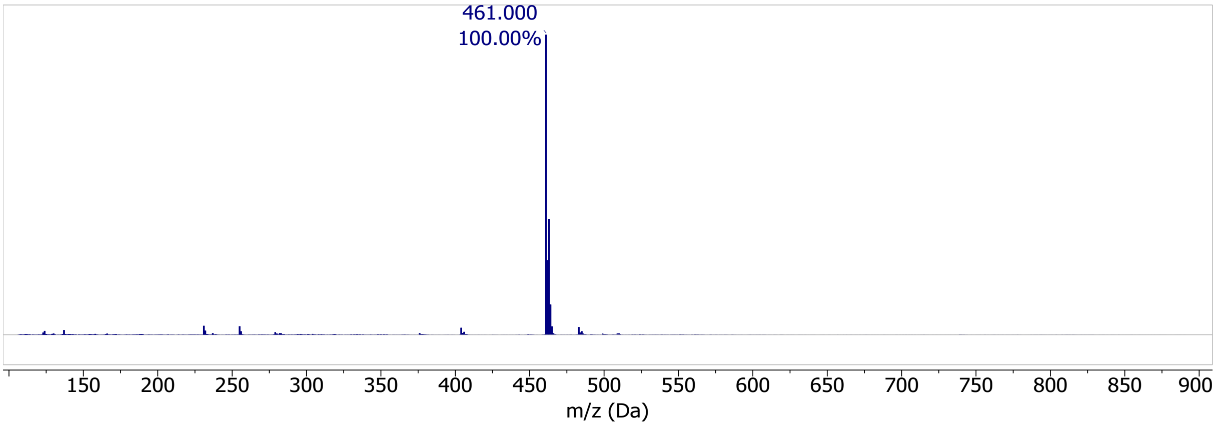

Cpd 6 (2A3, CW008)

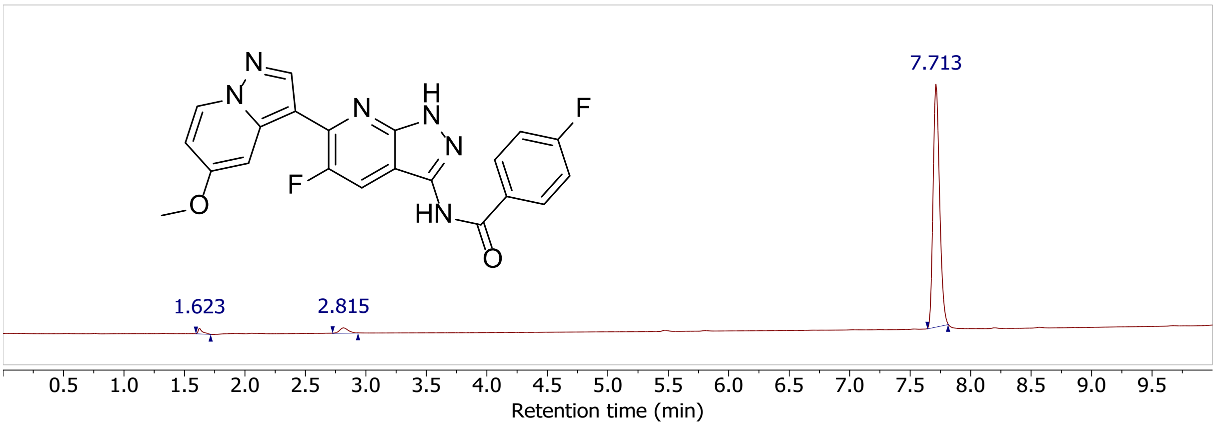

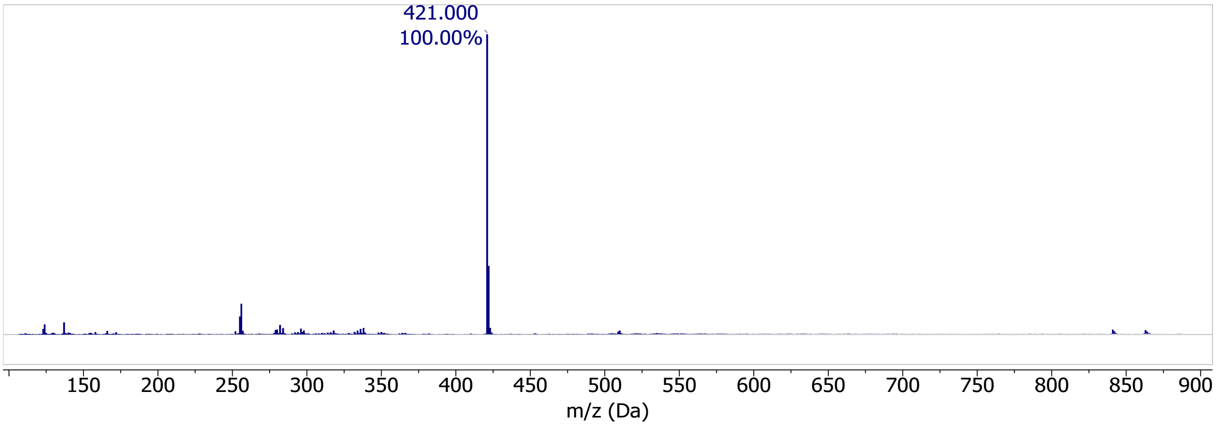

Cpd 7 (2D9, Autophinib)

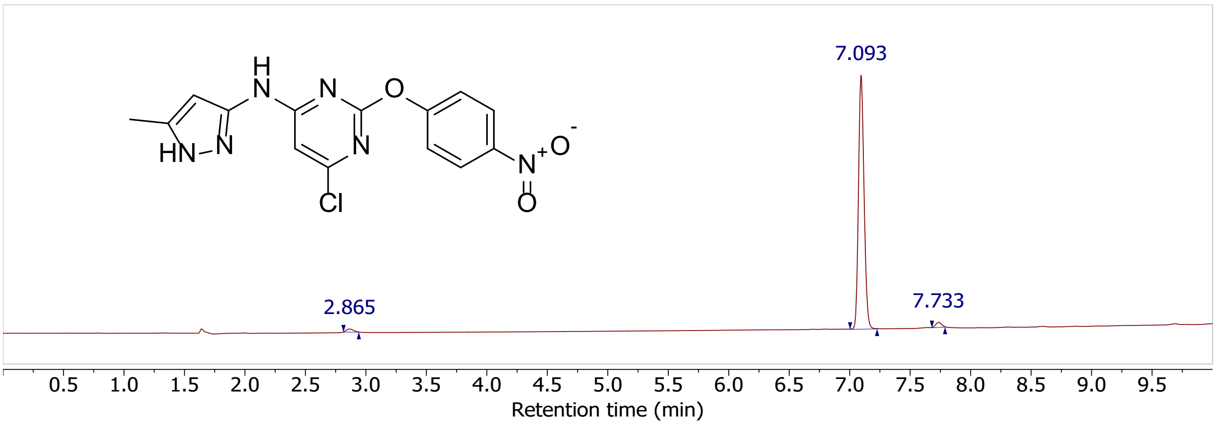

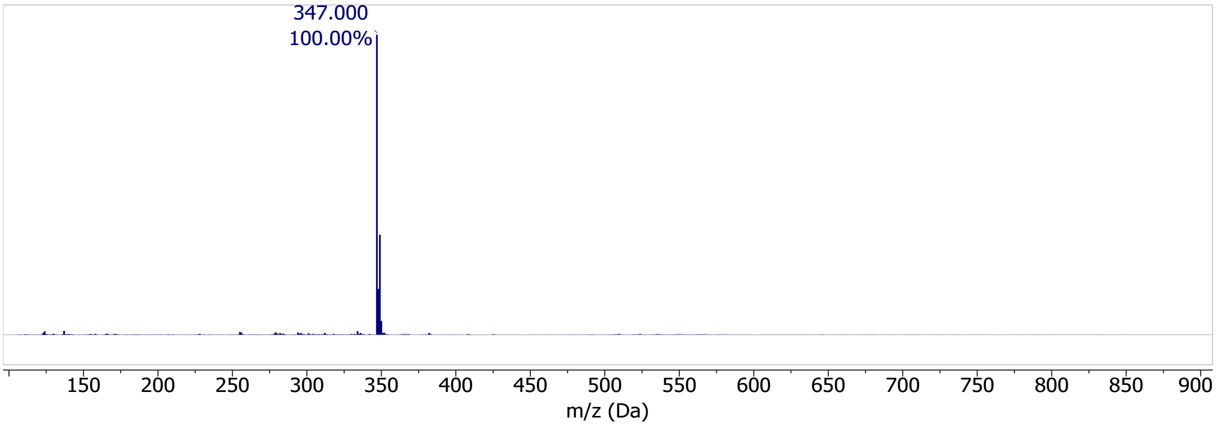

Cpd 8 (2G8, CRT 0066854)

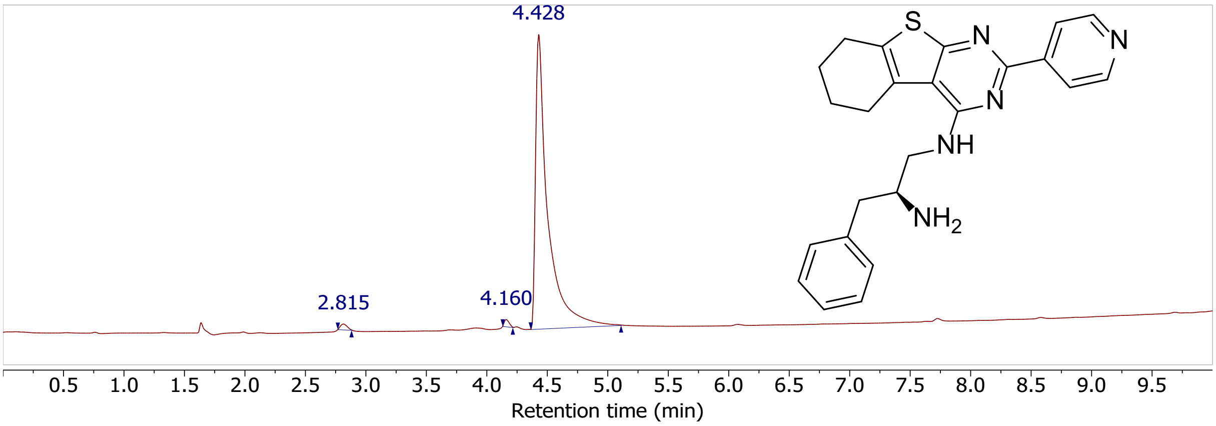

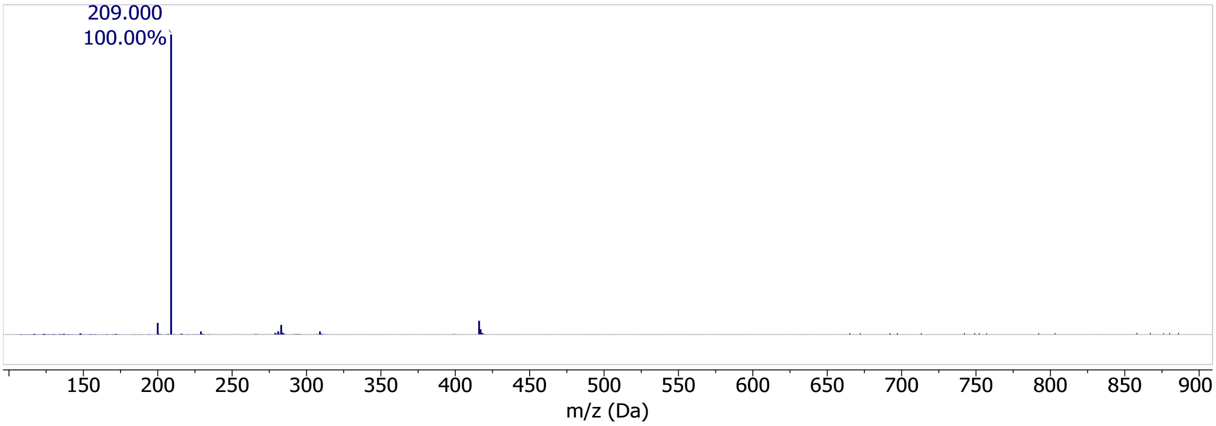

Cpd 9 (2E2, A 485)

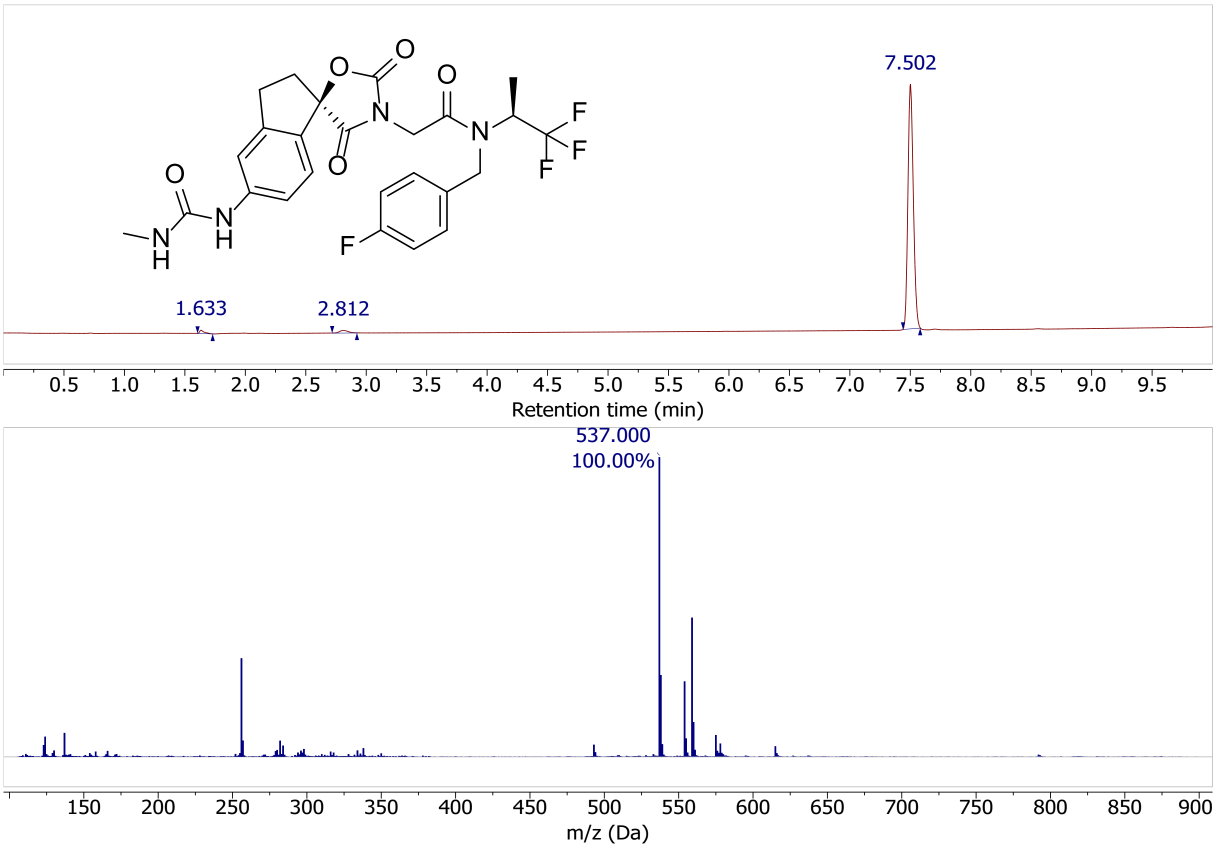

Cpd 10 (2E6, FM19G11)

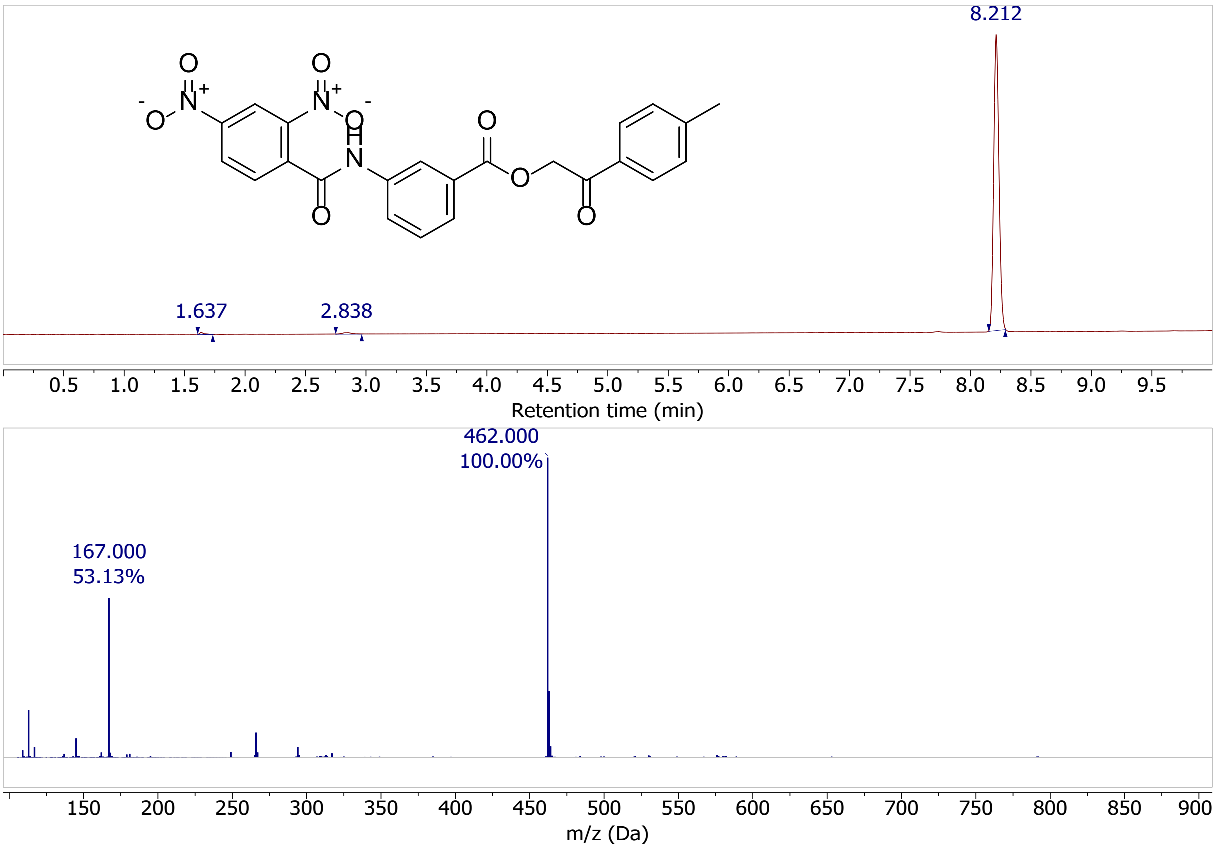

Cpd 11 (12B8, 1-(adamantan-1-yl)-3-(prop-2-enoyl)urea)

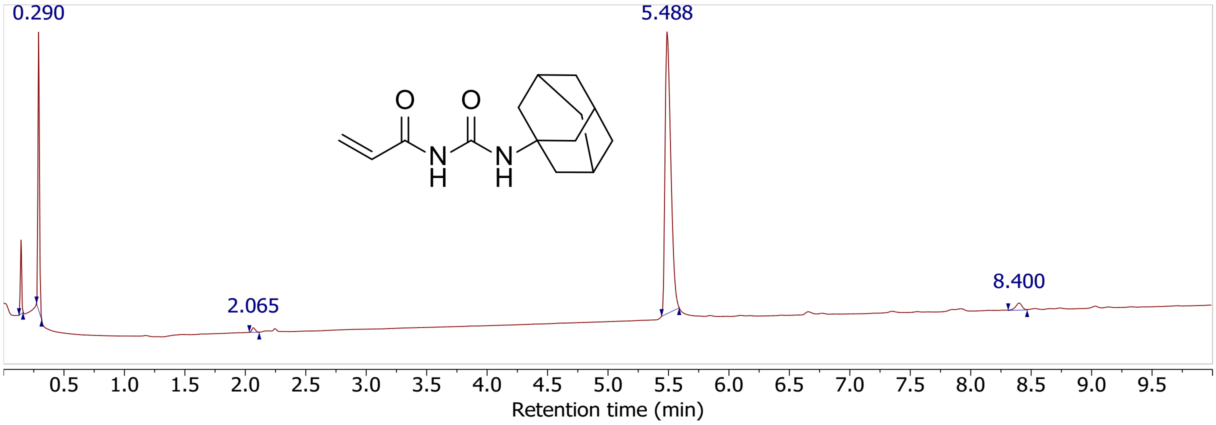

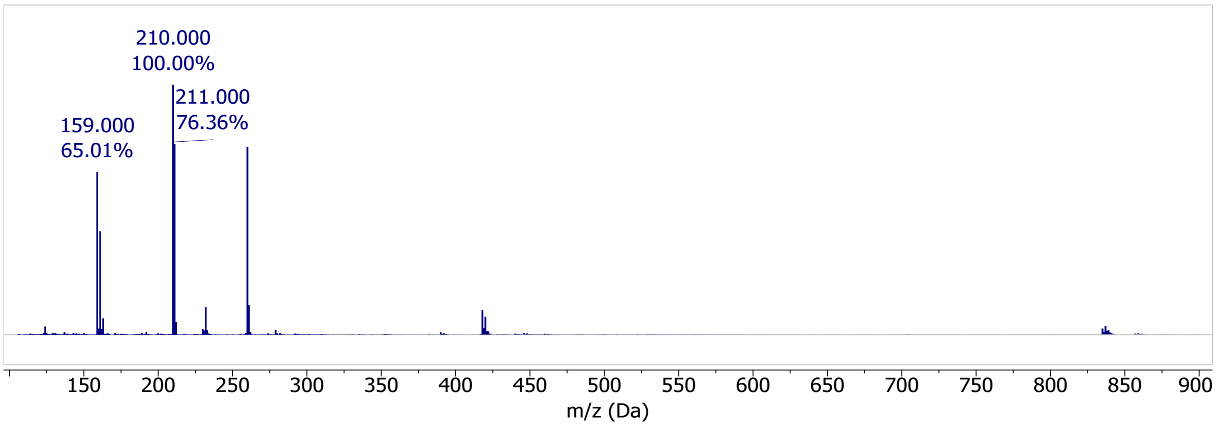

Supplement: Supplementary file 1 — Supplementary Information [file 42004_2025_1439_MOESM1_ESM.pdf]
